# Novel Lysosomal‐Associated Transmembrane Protein 4B‐Positive Stem‐Like Cell Subpopulation Characterizes High‐Risk Colorectal Cancer Subtypes

**DOI:** 10.1002/mco2.70284

**Published:** 2025-07-13

**Authors:** Yangyang Fang, Tianmei Fu, Ziqing Xiong, Qian Zhang, Wei Liu, Kuai Yu, Aiping Le

**Affiliations:** ^1^ Department of Transfusion Medicine, Key Laboratory of Jiangxi Province for Transfusion Medicine, The First Affiliated Hospital, Jiangxi Medical College Nanchang University Nanchang Jiangxi China

**Keywords:** colorectal cancer, leucine‐rich repeat‐containing G‐protein coupled receptor 5 (LGR5), lysosome‐associated transmembrane protein 4B (LAPTM4B), stem‐like cells

## Abstract

Colorectal cancer (CRC) exhibits substantial intertumoral heterogeneity, largely attributable to multiple tumor stem‐like cell populations, whose molecular identities and clinical significance remain incompletely defined. This study delineates tumor‐intrinsic stem‐like cell diversity and its prognostic implications through single‐cell transcriptomic profiling of 171,906 tumor epithelial cells (*n* = 152), integrated with bulk transcriptomic (*n* = 1389) and genomic (*n* = 1077) datasets. Functional validation was conducted via in vitro assays and multiplex immunofluorescence. A previously unrecognized lysosome‐associated transmembrane protein 4B‐positive (LAPTM4B^+^) stem‐like cell cluster was identified, distinct from the classical leucine‐rich repeat‐containing G‐protein coupled receptor 5‐positive (LGR5^+^) population. LAPTM4B^+^ cells exhibited MYC pathway activation and 8q chromosomal gains, with preferential enrichment in microsatellite‐stable, *POLE* wild‐type, and left‐sided tumors. Stratification based on LAPTM4B^+^/LGR5^+^ stem‐like cell ratios defined four CRC stem‐like subtypes (CSS), with CSS2 (LAPTM4B^+^‐dominant) associated with the poorest prognosis (HR = 2.31, *p* < 0.001). The combined expression of LAPTM4B and LGR5 demonstrated superior predictive power for CRC progression compared to either marker alone (AUC = 0.820 vs. 0.715/0.699), underscoring the synergistic influence of distinct stem‐like cell populations on patient outcomes. These findings provide novel insights into CRC heterogeneity and cooperative interactions among diverse stem‐like populations shaping disease outcomes.

## Graphical abstract

This study identified the existence of a novel population of LAPTM4B^+^ tumor stem‐like cells in addition to the classical LGR5^+^ tumor stem‐like cells in colorectal cancer (CRC). The combination of LGR5^+^ and LAPTM4B^+^ stem‐like cells enable a more refined stratification of CRC, offering potential insights for targeted therapeutic strategies.

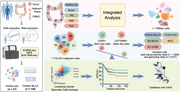

## 1 Introduction

Colorectal cancer (CRC) represents a formidable challenge in oncology, with surgical resection serving as the primary therapeutic strategy. However, despite advancements in surgical techniques, over 30% of patients develop metastases within a few years postsurgery [1]. CRC is characterized by profound heterogeneity [2], primarily reflected in aberrant cellular differentiation. Poorly differentiated tumor cells frequently acquire stem cell‐like properties [3], a major driver of intratumoral heterogeneity [4]. Accumulating evidence underscores the pivotal role of these stem‐like tumor cells in tumor initiation, progression, and metastasis [5, 6]. Thus, delineating intratumoral heterogeneity at the cellular level, particularly within stem‐like tumor cell populations, holds promise for refining CRC treatment strategies.

Over the past decade, investigations into CRC stem‐like cells have identified key markers and pathways. Seminal studies established leucine‐rich repeat‐containing G‐protein coupled receptor 5 (LGR5) as a canonical intestinal stem‐like cell marker [7], while subsequent research revealed heterogeneous stem‐like subsets expressing alternative surface markers such as CD133, CD44, and CD166 [8]. However, a reliance on surface marker‐based classification risks overlooking stem‐like populations defined by intracellular regulators. For instance, nuclear factors like ASCL2 drive stemness via WNT pathway activation, highlighting the limitations of surface marker‐centric approaches in capturing the full spectrum of stem‐like plasticity [9].

Single‐cell RNA sequencing (scRNA‐seq) has transformed the ability to resolve tumor heterogeneity by enabling unbiased transcriptional profiling, circumventing the constraints of surface marker‐dependent methods, and providing precision in dissecting tumor complexity [10]. Recent scRNA‐seq studies have identified enrichment of stem‐like malignant cells [11], tumor‐associated macrophages [12], and stromal cells [13] within metastatic sites, implicating these populations in the metastatic process. However, these studies remain constrained by limited cohort sizes and sample numbers, restricting comprehensive exploration of biological heterogeneity and its clinical relevance. Furthermore, the absence of genomic data impedes the integration of single‐cell transcriptomic alterations with underlying genetic variations.

To overcome these challenges, this study integrates scRNA‐seq with bulk RNA‐seq and whole‐exome sequencing (WES), identifying a previously unrecognized lysosome‐associated transmembrane protein 4B‐positive (LAPTM4B^+^) stem‐like population distinct from classical leucine‐rich repeat‐containing G‐protein coupled receptor 5‐positive (LGR5^+^) cells. LAPTM4B^+^ subsets exhibited 8q chromosomal gains and were enriched in microsatellite‐stable (MSS), DNA polymerase epsilon catalytic subunit A (*POLE*) wild‐type, and left‐sided CRCs. Bulk transcriptomic analyses across five independent cohorts classified CRC subtypes based on LAPTM4B^+^/LGR5^+^ stem‐like signatures, linking these subgroups to survival outcomes, transcriptional programs, and genomic profiles. Patients with predominant LAPTM4B^+^ stem‐like populations exhibited the highest risk of disease recurrence. Moreover, combined quantification of LAPTM4B and LGR5 expression demonstrated superior predictive power for tumor progression compared to individual markers, underscoring the synergistic influence of heterogeneous stem‐like cell populations on patient outcomes.

Collectively, these findings provide critical insights into CRC stem‐like cell heterogeneity and identify distinct stem‐like subpopulations that shape disease progression. This study advances the understanding of tumor heterogeneity and offers potential avenues for therapeutic stratification.

## 2 Results

### 2.1 Single‐Cell Transcriptomic Landscape of Colorectal Cancer

**FIGURE 1:**
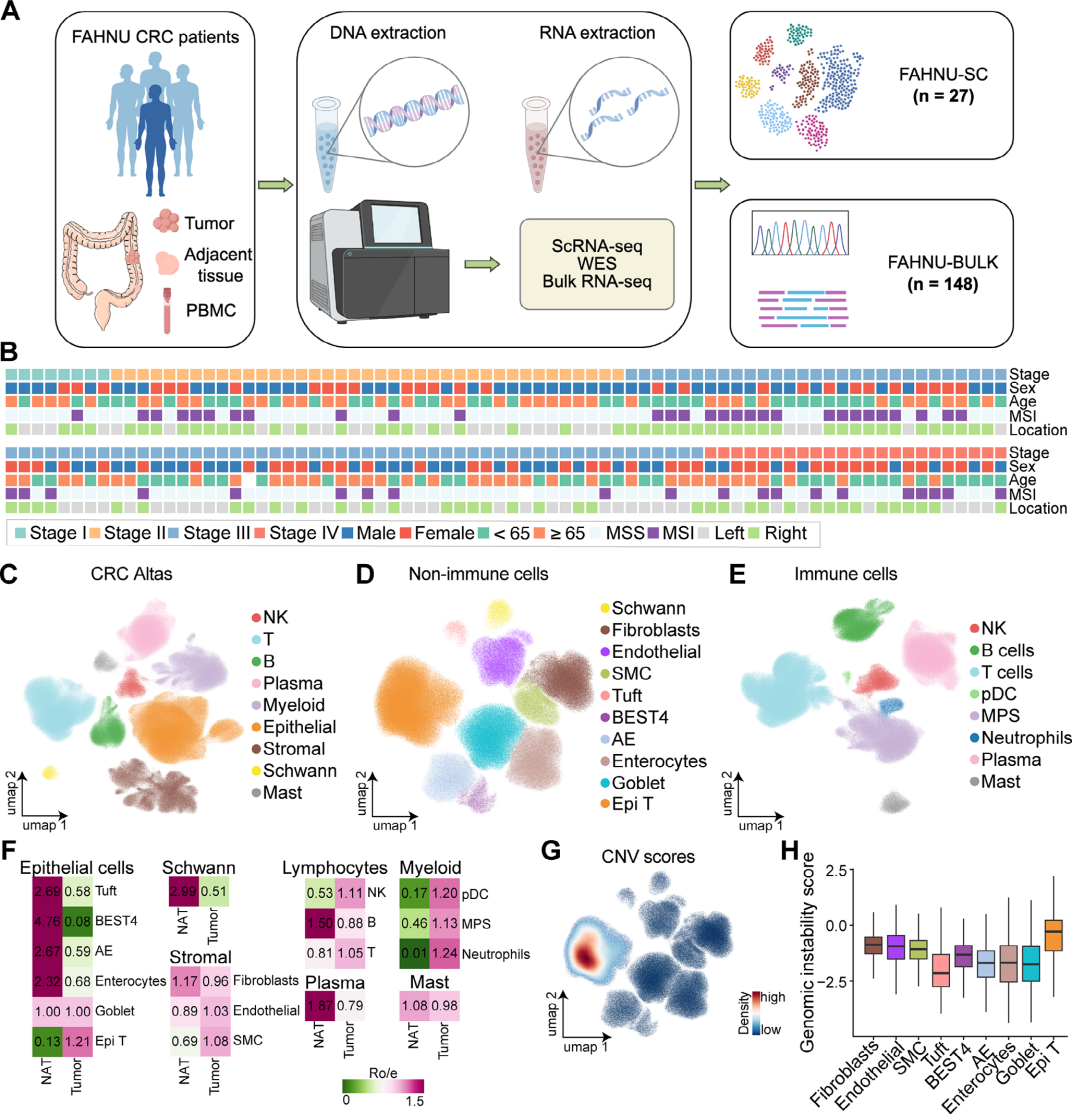
Establishment of the CRC single‐cell atlas. (A) A schematic flowchart delineates the sample collection process for the FAHNU‐SC and FAHNU‐BULK cohorts, specifically curated for CRC. (B) Demographic and clinical characteristics, including sex, age, microsatellite status, tumor stage, and anatomical tumor location, were compiled for 152 patients with CRC. (C) A UMAP projection visualizes 1,001,621 cells from these patients, classified into nine major cell types. (D, E) Distinct UMAP plots further delineate the clustering of nonimmune cells and immune cells within the dataset. (F) A heatmap quantifies the distribution bias of each cell subset between tumor tissue and normal adjacent tissue (NAT) based on the ratio of observed‐to‐expected ratio (Ro/e). (G) A UMAP plot highlights copy number variation (CNV) scores across all nonimmune cells. (H) A box plot presents genomic instability scores of nonimmune cells, reflecting chromosomal aberration levels.

WES and bulk RNA‐seq were performed on surgical resection samples from 148 patients with primary CRC. Additionally, scRNA‐seq was conducted on tumor tissues from 27 patients with CRC. The comprehensive analysis workflow is depicted in Figure 1A.

To further elucidate CRC heterogeneity, scRNA‐seq data from the FAHNU‐SC cohort (*n* = 27) were integrated with datasets from four independent scRNA‐seq studies, yielding a combined dataset of 152 patients with primary CRC, encompassing both tumor (*n* = 152) and adjacent normal tissues (*n* = 73). Clinical information, including age, pathological stage, tumor location, and microsatellite status, was available for most patients (Figure 1B and Table S1). Integration of single‐cell datasets was performed using scArches, followed by quality control and batch correction, resulting in a final dataset of 1,001,621 cells. Coarse cell type annotation identified nine major cell types: NK cells, T cells, B cells, plasma cells, myeloid cells, epithelial cells, stromal cells, Schwann cells, and mast cells (Figures 1C and S1B). Nonimmune populations were further classified into epithelial and stromal subtypes. The epithelial compartment included goblet cells, Bestrophin‐4 (BEST4) cells, absorptive enterocytes (AEs), enterocytes, tuft cells, and tumor epithelial (Epi T) cells, while stromal cells were categorized into fibroblasts, endothelial cells, and smooth muscle cells (SMCs, Figures 1D and S1A). Myeloid lineage immune cells were further subdivided into neutrophils, plasmacytoid dendritic cells (pDCs), and the mononuclear phagocyte system (MPS, Figures 1E and S1A).

Cell type proportions were analyzed across different datasets and tissue types. Among nonimmune cells, Schwann cells, AE, tuft cells, enterocytes, and BEST4 cells were predominantly enriched in adjacent normal tissues, whereas Epi T cells were almost exclusively confined to tumor tissues. Within the immune compartment, neutrophils and pDCs, both of myeloid origin, were enriched in tumor tissues (Figures 1F and S1C).

Consistent with previous findings, microsatellite instability (MSI) tumors exhibited a higher abundance of immune cells within tumor tissues, whereas MSS tumors showed a greater prevalence of stromal cells (Figure S1D). Regarding tumor location, left‐sided CRC demonstrated a higher proportion of stromal cells (Figure S1E). Among epithelial subsets, Epi T cells exhibited the highest copy number variation (CNV) scores (Figure 1G) and the greatest genomic instability scores (GISs; Figures 1H and S1F), reinforcing their malignant potential.

### 2.2 Epi T Cell Annotation

**FIGURE 2:**
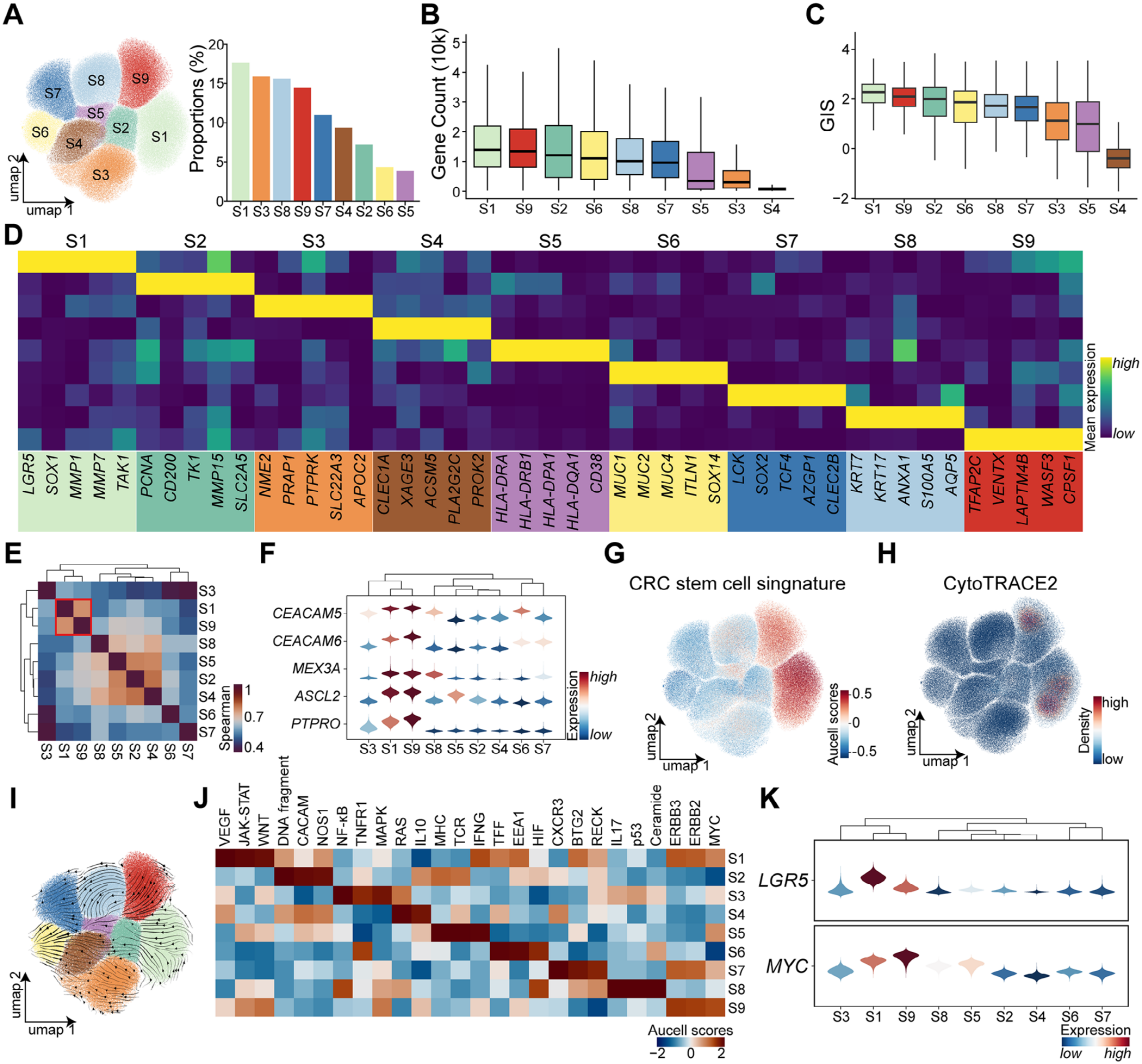
Features of tumor stem‐like cell subpopulations. (A) UMAP dimensionality reduction of 171,906 epithelial tumor (Epi T) cells, the cells are stratified into nine subsets (S1–S9), with a bar chart illustrating their relative proportions. (B) A box plot displays gene counts distributions across S1–S9 subsets. (C) Another box plot compares genomic instability scores among S1–S9 subsets. (D) A heatmap showcases marker genes specific to each subpopulation. (E) A correlation heatmap, derived from Spearman's analysis, assesses gene expression similarities among the nine subpopulations, with red indicating positive correlation and blue denoting negative correlation. (F) Violin plot highlights the shared high expression of specific genes within the S1 and S9 subsets. (G, H) Density plots depict AUCell scores for CRC stem‐like traits and CytoTRACE2 scores across the nine Epi T subsets. (I) RNA velocity streamlines embedded in the UMAP projection visualize transcriptional dynamics among the S1–S9 subpopulations. (J) A heatmap outlines enriched signaling pathways within the nine subsets. (K) Violin plots illustrate the expression patterns of *LGR5* and *MYC* within tumor stem‐like cells in the S1 and S9 subsets.

To delineate epithelial tumor cell subsets, 171,906 Epi T cells were classified into nine distinct clusters (S1–S9). Among these, S1 represented the most prevalent population, whereas S5 was the least abundant (Figure 2A). In single‐cell transcriptomics, gene expression levels often correlate with cellular differentiation potential [14]. Notably, S1 and S9 subsets exhibited the highest gene counts (Figure 2B), along with elevated GISs (Figure 2C). Marker gene analysis corroborated these findings, revealing that S1 cells highly expressed *LGR5*, a well‐established marker of intestinal cancer stem‐like cells. The remaining subsets displayed distinct molecular signatures: S2 cells were characterized by high *PCNA* expression, indicative of proliferative activity; S3 cells were marked by *NME2*; S4 cells by *CLEC1A*; S5 cells exhibited dual epithelial and immune properties, with *HLA‐DRA* and *HLA‐DRB1* expression; S6 cells expressed mucin family markers *MUC1*, *MUC2*, and *MUC4*; S7 cells were defined by *LCK*; S8 cells were characterized by *KRT7* and *AXAN1*; and S9 cells exhibited high expression of *TFAP2C* and *LAPTM4B* (Figure 2D).

Expression profiles similarity analysis revealed that S1 and S9 shared the most comparable transcriptional features (Figure 2E). Several oncogenes, including the oncogenes *CEACAM5* and *CEACAM6*, were upregulated in both subsets, alongside stemness‐associated genes such as *MEX3A* [15], *ASCL2* [15], and PTPRO [11] (Figure 2F). Stemness scoring via AUCell further confirmed that S1 and S9 subsets exhibited the highest stemness‐related gene signatures (Figure 2G and Table S2). Additionally, CytoTRACE2 predictions indicated that these subsets harbored the greatest differentiation potential (Figure 2H), and RNA velocity analysis suggested that differentiation trajectories originate from S1 and S9 cells toward other tumor epithelial subsets (Figure 2I).

Previous research has implicated MYC and WNT pathway hyperactivation in CRC stem‐like cells [11]. AUCell pathway analysis across all subsets demonstrated that both S1 and S9 cells exhibited elevated WNT and MYC pathway activity. However, WNT overactivation was more pronounced in S1, whereas S9 cells predominantly exhibited MYC activation (Figure 2J). Both *LGR5* and *MYC*, markers of stem‐like characteristics, were highly expressed in these subsets, with S1 showing stronger *LGR5* expression and S9 displaying higher *MYC* expression (Figure 2K).

Metabolic profiling revealed that S1 and S9 cells share similar metabolic characteristics, both exhibiting heightened metabolic activity (Figure S2). These results indicate the presence of two highly similar yet distinct stem‐like cell populations in CRC, each characterized by unique activation pathways and functional properties. Cell–cell interaction analysis demonstrated that S9 cells engaged in slightly more interactions than S1 cells and exhibited active communication with stromal cells (Figure S3A–C). Compared to S1 cells, S9 cells specifically participated in endothelial cell‐mediated CD39 signaling input and generated oncogenic LIFR signaling as an output (Figure S3D) [16].

### 2.3 LAPTM4B as a Marker Gene of the S9 Cell Subsets

**FIGURE 3:**
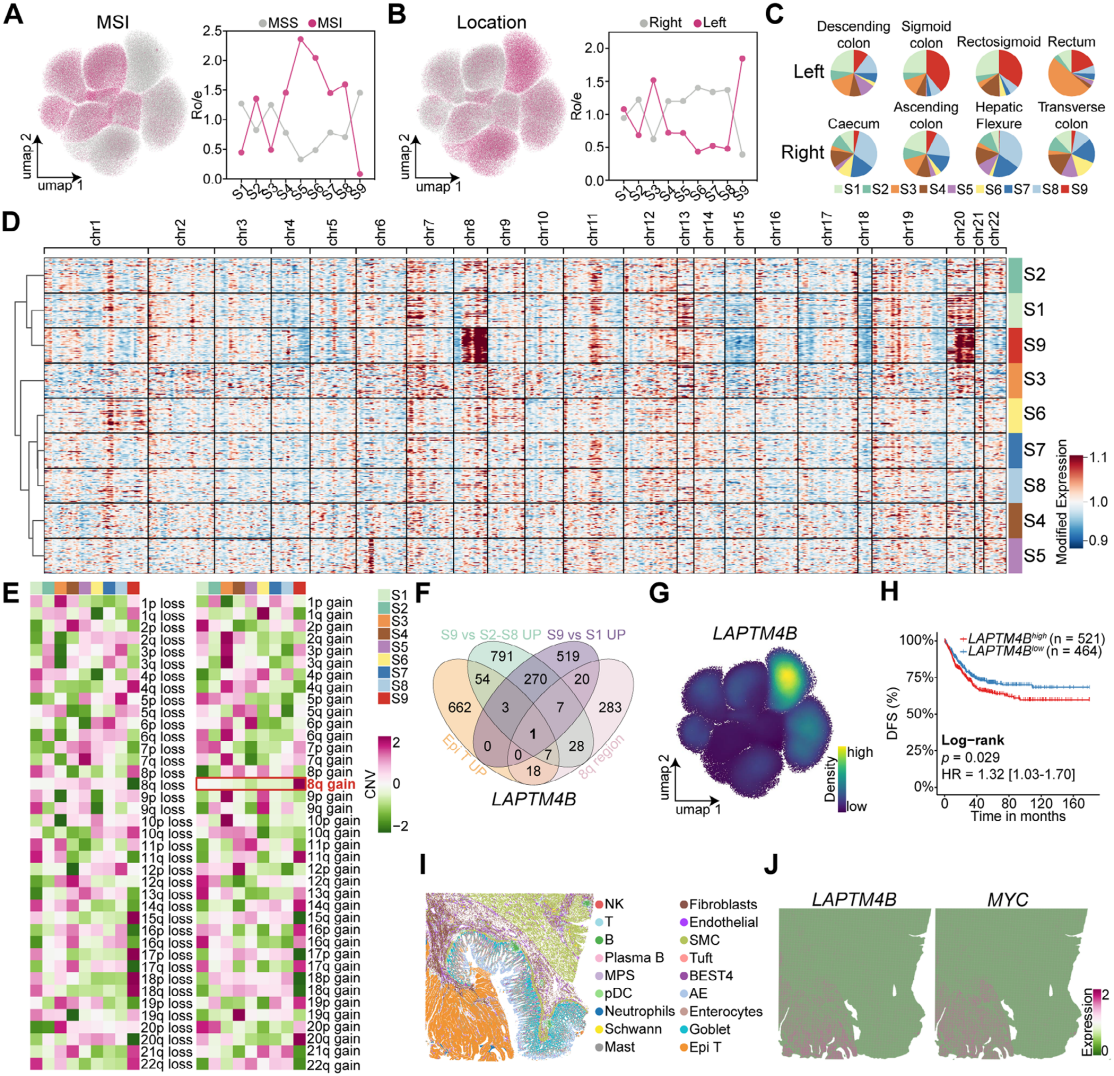
LAPTM4B as a marker gene for the S9 stem‐like cell subsets. (A) UMAP projection of epithelial tumor (Epi T) cells differentiates MSI (pink) from MSS (gray) tumors (left panel), while the right line graph quantifies the distribution biases of each cell subgroup based on MSI/MSS status. (B) Distribution patterns of Epi T subgroups according to tumor location. (C) Pie charts illustrating the proportional composition of various Epi T subsets across different tumor sites. (D) CNV profiles of S1–S9 subsets. (E) Heatmap summarizing copy number alterations across all 22 chromosomes in the S1–S9 subsets. (F) Venn diagram depicting the overlap between highly expressed genes in the S9 subset and genes located in the 8q chromosomal region (logFC > 2, FDR < 0.01). (G) UMAP projection mapping *LAPTM4B* expression in tumor epithelial cells. (H) Kaplan–Meier survival analysis stratified by *LAPTM4B* expression, with DFS assessed via the log‐rank test to evaluate prognostic significance (*n* = 985). (I) Integration of the CRC single‐cell atlas with spatial transcriptomic data. (J) Spatial transcriptomic visualization of *LAPTM4B* and *MYC* expression in CRC.

Further characterization of these stem‐like populations showed that both S1 and S9 subsets were predominantly enriched in MSS patients, with S9 cells being almost exclusively present in this subgroup (Figures 3A and S4A). Spatially, S9 cells, along with the S3 cell population, were primarily localized in left‐sided CRC, whereas S6, S7, and S8 cells were predominantly found in right‐sided tumors, S1 cells, in contrast, exhibited no distinct spatial preference (Figures 3B,C and S4A).

CNV profiling, inferred using inferCNV with five normal epithelial cell types as references, revealed that S1 and S9 subsets shared the most similar CNV patterns (Figure 3D). Notably, S9 cells exhibited a distinct amplification in the 8q region (Figure 3E), a feature absents in other epithelial subpopulations. Differential expression analysis identified key marker genes of the S9 subsets by selecting genes specifically upregulated in Epi T cells. Genes enriched in S9 relative to S2–S8 subsets, genes elevated in S9 compared to S1, and overlapping genes located within the 8q region (Figure 3F). This approach identified *LAPTM4B* as a defining marker of S9 cells, with high expression specifically within this subset (Figure 3G).

Bulk transcriptomic analysis corroborated these single‐cell findings, showing significant overexpression of *LAPTM4B* in left‐sided CRC (*p* < 0.0001; Figure S4B). Moreover, *LAPTM4B* was elevated in patients who were *POLE* wild type (*p* < 0.0001), younger (*p* < 0.0005), and in advanced disease stages (*p* < 0.001; Figure S4C–E). Prior studies by the CRC Subtyping Consortium defined four consensus molecular subtypes (CMSs) [17], with *LAPTM4B* being lowest in CMS1 (predominantly MSI‐enriched) and highest in CMS2 and CMS4, both characterized by high chromosomal instability (*p* < 0.0001; Figure S4F).

Survival analysis demonstrated that patients with high *LAPTM4B* expression had significantly shorter disease‐free survival (DFS; hazard ratio [HR] = 1.32; 95% confidence interval [CI] 1.03–1.70; *p* = 0.029; Figure 3H) and overall survival (OS; HR = 1.35; CI, 1.05–1.75; *p* = 0.021; Figure S4G) compared to those with low expression. Multivariable Cox regression analysis, incorporating tumor epithelial and stromal/immune infiltration scores as covariates, confirmed *LAPTM4B* overexpression as an independent prognostic factor for poor OS (HR = 1.42; CI, 1.09–1.86, *p* = 0.009; Figure S4H) and DFS (HR = 1.31; CI, 1.01–1.69, *p* = 0.039; Figure S4I) after adjusting for cellular composition variations across samples. Spatial transcriptomics analysis further revealed that *LAPTM4B* and *MYC* were highly expressed in Epi T cells (Figures 3I,J and S4J,K), reinforcing their role in CRC progression.

Both tumor stem‐like cell populations were predominantly localized to tumor tissues, and only sparse LAPTM4B^+^ cells were detectable in normal intestinal tissues (Figure S5A). Among nonmalignant epithelial cells, *LAPTM4B* exhibited low‐level expression in Tuft cells. Coclustering analysis confirmed transcriptional signature similarities between Tuft cells and the two stem‐like clusters (Figure S5B,C).

### 2.4 LAPTM4B Identifies Stem‐Like Cells With High c‐Myc Expression in MSS CRC

**FIGURE 4:**
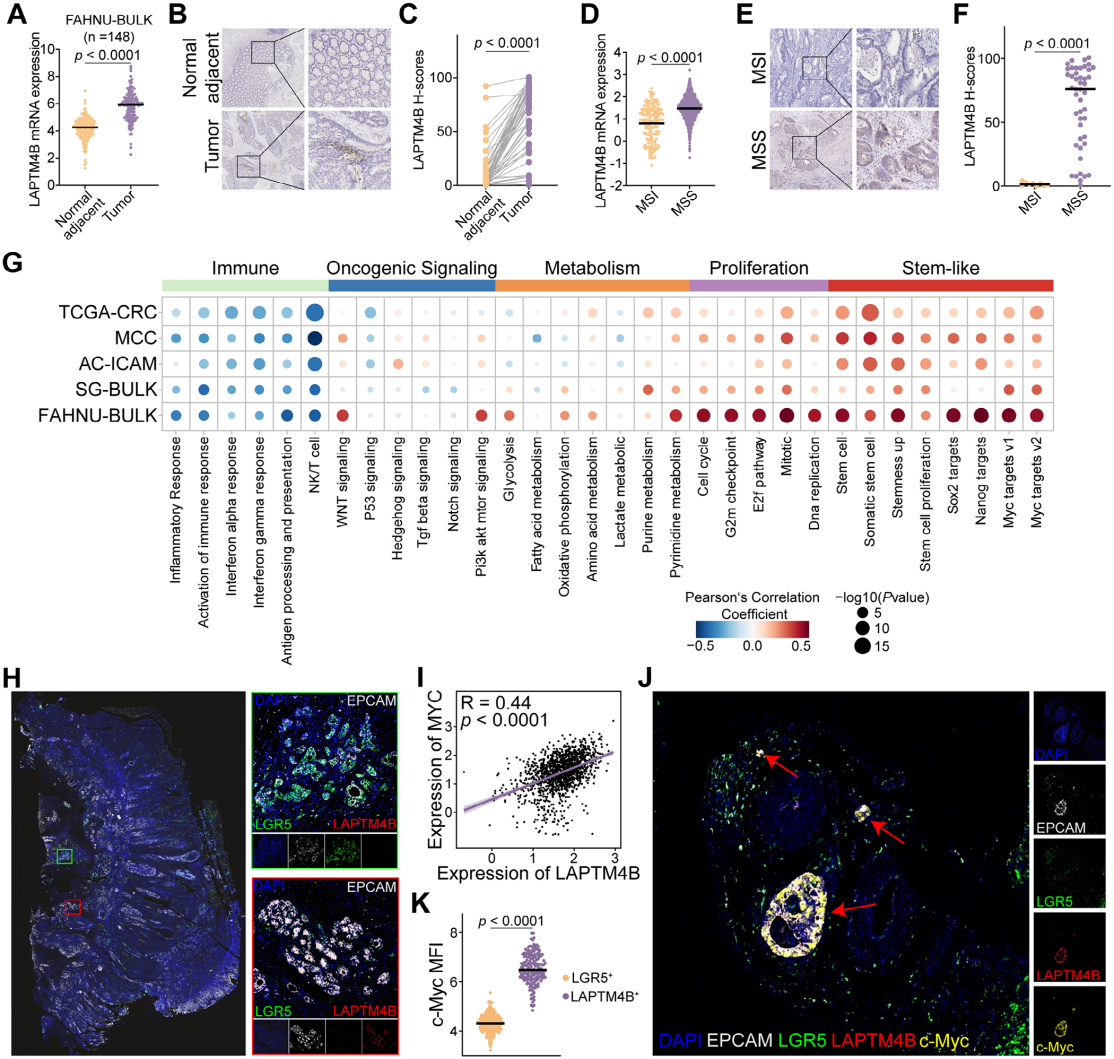
LAPTM4B identifies stem‐like cells with high c‐Myc expression in MSS CRC. (A) *LAPTM4B* mRNA levels in CRC tumor tissues versus paired normal adjacent tissues from the FAHNU‐BULK cohort (*n* = 148). (B) Representative IHC images of LAPTM4B staining in tumor and adjacent normal tissues from 47 patients with CRC. (C) Quantitative analysis of LAPTM4B protein expression in CRC tumors versus normal tissues via IHC. (D) *LAPTM4B* mRNA expression in MSI and MSS tumors. (E) Representative IHC images showing LAPTM4B expression in MSI and MSS tumors. (F) Quantification of LAPTM4B expression in MSI (*n* = 9) versus MSS (*n* = 46) tumors via IHC. (G) Bubble plot depicting pathways associated with *LAPTM4B*, where red indicates positive correlation, blue denotes negative correlation, and bubble size represents *p* value significance. (H) Representative mIHC image of a CRC tumor section displaying DAPI^+^ (blue), EPCAM^+^ (white), LGR5^+^ (green), and LAPTM4B^+^ (red) cells. (I) Scatter plot illustrating the correlation between *LAPTM4B* and *MYC* mRNA expression (*R* = 0.44, *p* < 0.0001). (J) Representative mIHC image showing EPCAM^+^ (white), LGR5^+^ (green), LAPTM4B^+^ (red), and c‐Myc^+^ (orange) cells, with nuclei counterstained by DAPI (blue). (K) Mean fluorescence intensity (MFI) of c‐Myc in LGR5^+^ and LAPTM4B^+^ stem‐like tumor cells, with statistical significance (*p* value) determined by a two‐sided *t*‐test.

Bulk RNA‐seq analysis of the FAHNU‐BULK cohort (*n* = 148) revealed significantly elevated *LAPTM4B* expression in tumor tissues compared to adjacent normal tissues (*p* < 0.0001; Figure 4A). These observations were further validated by immunohistochemistry (IHC), which demonstrated a pronounced increase in LAPTM4B expression within tumor samples (*p* < 0.0001; Figure 4B,C). Consistent with scRNA‐seq data, MSI patients exhibited lower LAPTM4B expression levels than those with MSS (*p* < 0.0001; Figure 4D–F).

To elucidate the regulatory networks associated with *LAPTM4B*, pathway enrichment analysis was performed using gene sets from Molecular Signatures Database (MsigDB) across five independent CRC cohorts. *LAPTM4B* expression showed a negative correlation with immune‐related pathways, including inflammatory response, immune response activation, and NK/T cell pathways, while displaying a strong positive association with cell proliferation pathways. Notably, *LAPTM4B* was highly correlated with stemness‐associated pathways, such as Stemness Up, Sox2 targets, Nanog targets, and Myc targets v1/v2. Among these, the Myc targets pathway exhibited the strongest positive correlation with *LAPTM4B* across all five cohorts (Figure 4G).

Multiplex IHC (mIHC) on formalin‐fixed, paraffin‐embedded (FFPE) CRC samples demonstrated that *LAPTM4B* ^+^ stem‐like cells and *LGR5* ^+^ stem‐like cells coexisted within CRC tissues, albeit as distinct cellular subsets (Figure 4H). Pearson correlation analysis revealed a significant positive correlation between *LAPTM4B* and *MYC* (*R* = 0.44; *p* < 0.0001; Figure 4I). Further investigation using immunofluorescence staining showed that c‐Myc expression was markedly upregulated in LAPTM4B^+^stem‐like cells compared to LGR5^+^ stem‐like cells (*p* < 0.0001; Figure 4J,K). These results suggest that LAPTM4B^+^ stem‐like cells represent a distinct subset of c‐Myc‐high‐expressing CRC stem‐like cells, predominantly enriched in MSS patients.

### 2.5 Knockdown of LAPTM4B Inhibits Stemness in CRC Tumor Cells

**FIGURE 5:**
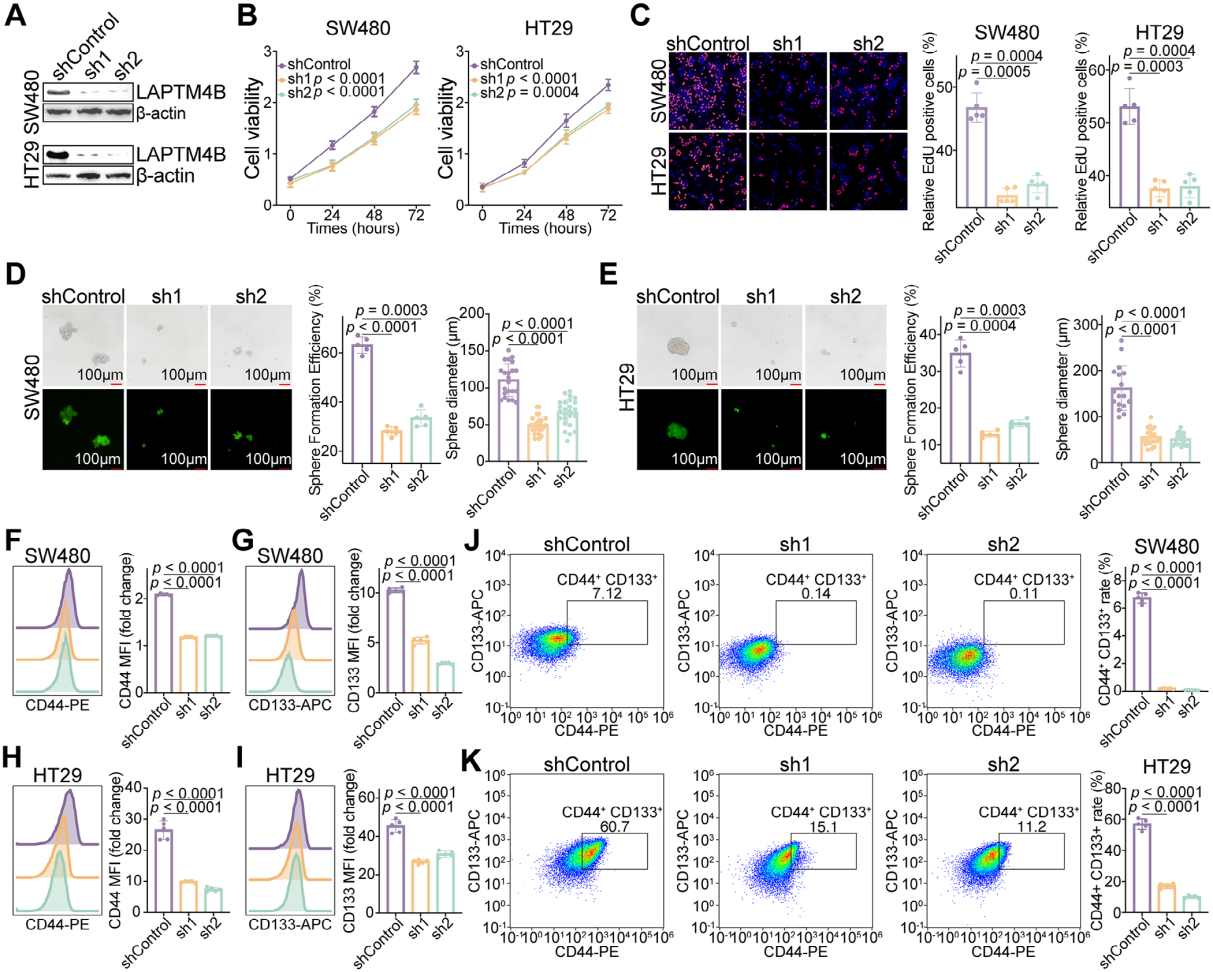
LAPTM4B knockdown suppresses stemness in CRC tumor cells. (A) Western blot analysis confirming LAPTM4B knockdown. (B, C) CCK‐8 and EdU assays demonstrating the inhibitory effect of LAPTM4B knockdown on CRC cell proliferation. (D, E) Reduced spheroid formation capacity in SW480 and HT29 cells following LAPTM4B knockdown. (F–I) Decreased MFI of CD44‐PE and CD133‐APC in SW480 and HT29 cells after LAPTM4B suppression. (J, K) Representative flow cytometry dot plots quantifying CD44^+^CD133^+^ cell populations post‐LAPTM4B knockdown. Statistical significance (*p* value) was assessed using two‐sided *t*‐tests (*n* = 5 technical replicates per sample).

Across multiple CRC cohorts, *LAPTM4B* expression was positively correlated with tumor proliferation and stemness. To further validate its functional role, LAPTM4B knockdown was performed in two CRC cell lines (Figures 5A and S6A). Suppression of LAPTM4B significantly inhibited CRC cell proliferation (Figure 5B,C), with a marked reduction in both the number and size of spheroid formations compared to controls (Figure 5D,E).

Flow cytometry analysis further revealed that LAPTM4B knockdown significantly downregulated the expression of CRC stem cell markers CD44 and CD133 (Figure 5F,G). Given that CD44^+^CD133^+^ stem‐like cells are associated with heightened malignancy and oncogenic potential [18], LAPTM4B knockdown also led to substantial impairment in the generation of CD44^+^CD133^+^ cells (Figures 5J,K and S6B). These results collectively indicate that LAPTM4B is essential for maintaining the stem‐like properties of CRC tumor cells.

### 2.6 Intersection of LGR5^+^ and LAPTM4B^+^ Stem‐Like Cells Define CRC Stratification

**FIGURE 6:**
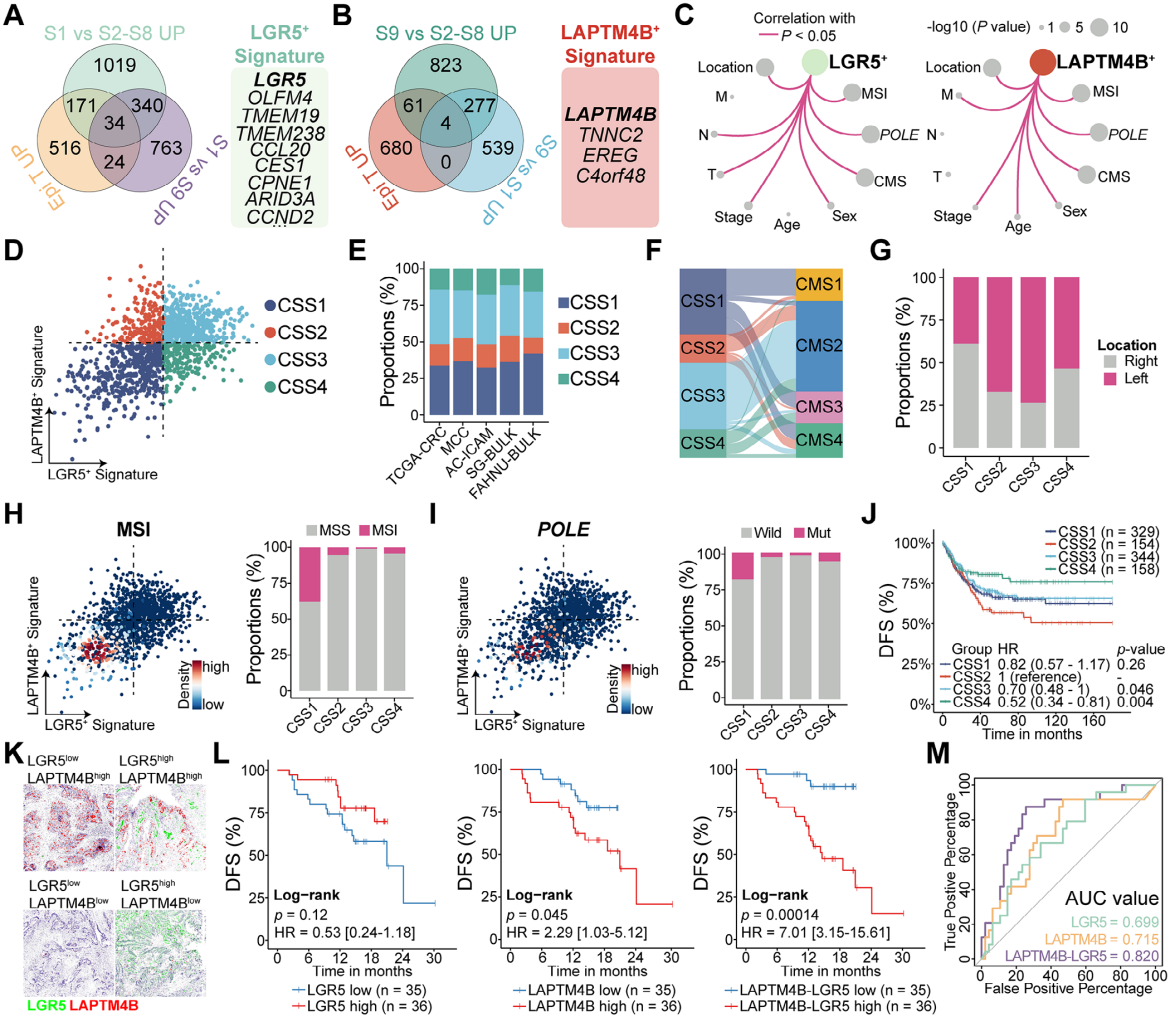
Intersection of LGR5^+^ and LAPTM4B^+^ stem‐like cells define CRC stratification. (A, B) Venn diagrams illustrating gene signatures and the number of genes identified in LGR5^+^ (A) and LAPTM4B^+^ (B) tumor stem‐like cells. (C) Network plot depicting correlation analysis between clinical molecular features and stem‐like cell signatures, with node size representing −log(*p* value). A *p* value < 0.05 was considered statistically significant. (D) Scatter plot displaying median expression levels of LGR5^+^ and LAPTM4B^+^ tumor stem‐like cell signatures in individual CRC samples. CRC stem‐like cell subtypes (CSS) were assigned based on the relative expression of these two signatures. (E) Bar chart illustrating the relative proportions of CSS subtypes across five CRC cohorts. (F) Alluvial plot demonstrating the correspondence between CSS and consensus molecular subtypes (CMSs). (G) Bar chart showing the distribution of left‐sided (pink) and right‐sided (gray) tumors across the four CSS subtypes. (H, I) Distribution of MSI (H) and *POLE*‐mutant (I) tumors among the four CSS subtypes. (J) Kaplan–Meier curves for disease‐free survival (DFS) across CSS subtypes (*n* = 985). (K) Representative multiplex IHC (mIHC) images from 71 CRC tumor samples showing LGR5^+^ (green) and LAPTM4B^+^ (red). (L) Kaplan–Meier analysis for DFS based on LGR5 expression (left), LAPTM4B expression (middle), and combined LAPTM4B‐LGR5 expression (right). (M) Receiver operating characteristic (ROC) curves illustrating the predictive performance of LGR5, LAPTM4B, and combined LAPTM4B‐LGR5 protein levels in primary CRC for tumor recurrence. The area under the curve (AUC) for LAPTM4B‐LGR5 is 0.820. Statistical significance in survival analyses was determined using the log‐rank test.

To delineate the gene expression profiles characteristic of LGR5^+^ and LAPTM4B^+^ stem‐like cells, differential analyses were conducted across multiple groups. Unique transcriptional signatures for each stem‐like cell type were defined, identifying a 34‐gene signature for LGR5^+^ stem‐like cells, which includes *LGR5*, *OLFM4*, *TMEM19*, *TMEM238*, and *CCL20*, among others. The LAPTM4B^+^ stem‐like cell signature comprises *LAPTM4B*, *TNNC2*, *EREG*, and *C4orf48* (Figure 6A,B and Table S3).

The LGR5^+^ stem‐like cell signature exhibited significant associations with MSI status, *POLE* mutations, and tumor staging. Higher signature scores were observed in MSS and *POLE* wild‐type tumors, particularly in early‐stage lesions, independent of metastatic status (M). Similarly, the LAPTM4B^+^ stem‐like cell signature was elevated in MSS and *POLE* wild‐type cases. However, in contrast to the LGR5^+^ signature, the highest LAPTM4B^+^ signature scores were detected in Stage IV tumors with metastatic dissemination (Figures 6C and S6C–E).

To assess whether CRC tumors could be classified based on the relative expression of LGR5^+^ and LAPTM4B^+^ stem‐like cell signatures, median expression levels were calculated for each sample. Using the median as a threshold, Tumors were stratified into four distinct CRC stem‐like subtypes (CSS1, CSS2, CSS3, and CSS4) based on signature magnitude, a classification consistently reproduced across independent cohorts (Figure 6D,E). CSS1 tumors, predominantly composed of CMS1 cases, exhibited low scores for both signatures. CSS2 tumors were defined by a dominant LAPTM4B^+^ signature, whereas CSS3 tumors, primarily CMS2, displayed high scores for both stem‐like signatures. CSS4 tumors were characterized by a predominant LGR5^+^ signature (Figures 6F and S6F). Notably, CSS2 (67%) and CSS4 (73%) tumors were predominantly localized to the left side of the CRC (Figures 6G and S6G), while MSI and *POLE‐*mutated tumors were primarily concentrated in the CSS1 subtype (Figure 6H,I).

The association between CRC subtypes and clinical outcomes was further examined, revealing distinct differences in recurrence risk. Patients with CSS2 tumors, characterized by a predominance of LAPTM4B^+^ stem‐like cells, exhibited the shortest median DFS. In contrast, CSS4 tumors, enriched in LGR5^+^ stem‐like cells, were associated with the longest DFS. Recurrence risk in CSS1 and CSS3 tumors, where both stem‐like cell populations were balanced, was intermediate relative to CSS2 and CSS4 (Figure 6J). A similar trend was observed in the OS analysis (Figure S6H).

mIHC was performed to simultaneously assess LGR5 and LAPTM4B expression in 71 CRC tumor samples (Figure 6K). Consistent with previous studies, elevated LGR5 expression correlated with a reduced risk of recurrence (HR = 0.53; CI, 0.24–1.18; *p* = 0.12), whereas higher LAPTM4B expression was associated with poorer DFS (HR = 2.29; CI, 1.03–5.12; *p* = 0.045). Notably, the relative expression ratio of LAPTM4B to LGR5 demonstrated superior predictive accuracy for CRC recurrence (HR = 7.01; CI, 3.15–15.61; *p* = 0.00014; Figure 6L). Receiver operating characteristic (ROC) curve analysis further validated the prognostic value of this ratio, with the area under the curve (AUC) confirming its predictive capability (Figure 6M, AUC = 0.82). These results underscore the critical role of LGR5^+^ and LAPTM4B^+^ stem‐like cell proportions in determining CRC recurrence risk.

### 2.7 Genomic Features and Functional Associations of CRC Stem‐Like Cell Subtypes

**FIGURE 7:**
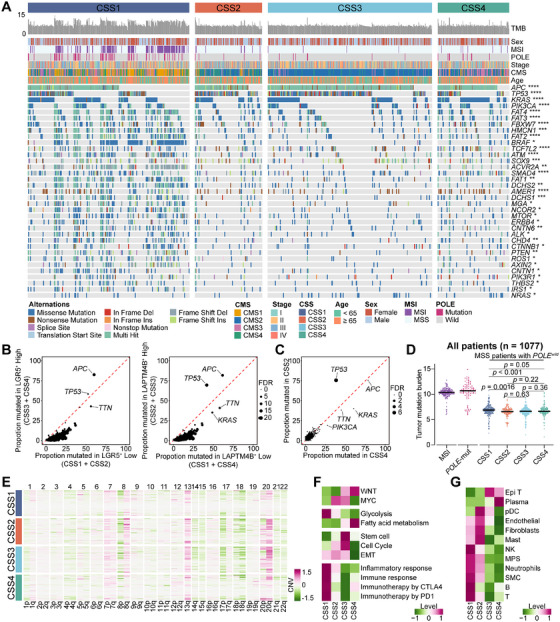
Genomic features and functional associations of CRC stem‐like cell subsets. (A) Overview of clinical and molecular characteristics across the four CSS subtypes. The top panel displays CSS classification, tumor mutational burden (TMB), sex, MSI status, *POLE* mutation status, pathological stage, CMS classification, and age. The bottom panel highlights 35 significantly different nonsynonymous mutations across the subtypes, with mutation types color‐coded and *p* values calculated using a two‐sided Fisher's exact test. (B) Scatter plots comparing mutation frequencies of genes in CSS3 + CSS4 versus CSS1 + CSS2 (left) and CSS2 + CSS3 versus CSS1 + CSS4 (right). (C) Scatter plots illustrating mutation frequencies of genes in CSS2 and CSS4, with point sizes corresponding to the BH‐corrected *q* values from two‐sided Fisher's exact tests. (D) TMB across CSS subtypes, with separate calculations for MSI and *POLE*‐mutant patients. (E) Chromosomal arm copy number variations (CNVs) from 1067 patients, stratified by CSS1, CSS2, CSS3, and CSS4. (F) Heatmap of GSVA enrichment scores for tumor‐associated signaling pathways across the four subtypes. (G) Heatmap displaying the average infiltration levels of 12 major tumor‐associated cell types across CSS subtypes, inferred from bulk transcriptomic data. All pairwise comparison *p* values were calculated using two‐sided *t*‐tests.

The relationship between two stem‐like cell populations and genomic alterations was examined in CRC. Genomic data from 1077 tumors were analyzed, identifying the 15 most frequently mutated genes across four patient subgroups, as shown in Figure S7A. Tumors classified as CSS3 and CSS4, enriched in LGR5^+^ stem‐like cells, exhibited higher *APC* mutation rates (84% and 78%, respectively). Given that *APC* mutations or functional loss led to aberrant activation of the WNT signaling pathway, this finding aligns with established mechanistic insights. CSS2 tumors demonstrated the highest *TP53* mutation rate (75%), while both CSS2 and CSS3 tumors, characterized by LAPTM4B^+^ stem‐like cell enrichment, displayed elevated mutation frequencies in both *APC* and *TP53* (Figure 7A,B).

To minimize confounding effects, CSS3 tumors, which harbor both stem‐like cell populations, were excluded from further comparative analyses. A direct comparison between CSS2 tumors, dominated by LAPTM4B^+^ stem‐like cells, and CSS4 tumors, primarily composed of LGR5^+^ stem‐like cells, was conducted. Notably, these two subtypes correspond to patient groups with the worst and best prognoses, respectively. The analysis revealed that *TP53* mutation was the only significantly enriched alteration in CSS2 tumors (Figures 7C and S7B). Furthermore, in tumors harboring *TP53* mutations, both the LAPTM4B^+^ stem‐like cell signature and *LAPTM4B* mRNA expression were markedly elevated compared to *TP53* wild‐type tumors (*p* < 0.0001; Figure S7C).

CSS1 tumors exhibited a significantly higher mutation burden than the other three subtypes, consistent with the predominance of MSI and hypermutator phenotypes in this group. Notably, even after excluding MSI and *POLE*‐mutated cases, CSS1 tumors retained a significantly elevated mutation burden compared to CSS2, CSS3, and CSS4, among which no significant difference in mutation burden were observed (Figures 7D and S7D).

CNV analysis revealed distinct genomic alterations across subtypes. CSS2 and CSS3 tumors exhibited recurrent gains in the 8q region, with the highest levels detected in CSS2, the subtype associated with poorest prognosis, exhibited the highest levels of 8q gain (*p* < 0.0001; Figures 7E and S7E). This observation aligns with findings from scRNA‐seq analysis, further supporting the prognostic relevance of 8q amplification in CRC.

Pathway enrichment analysis identified distinct molecular profiles among subtypes. CSS1 tumors exhibited the highest glycolysis activity and the lowest stem cell pathway activation while showing significant enrichment in immune‐related pathways. In contrast, CSS2 tumors were predominantly characterized by MYC pathway activation. CSS3 tumors displayed concurrent enrichment in the MYC and WNT pathways, along with heightened activation of stem cell pathways and cell cycle pathways, reflecting the coexistence of multiple stem‐like cell populations. CSS4 tumors were primarily enriched in the WNT and fatty acid metabolism pathways, exhibiting the lowest cell cycle activity and epithelial–mesenchymal transition (EMT) levels (Figure 7F). Tumor microenvironment analysis further delineated subtype‐specific immune and stromal compositions. CSS1 tumors exhibited elevated immune cell infiltration, including T cells, B cells, NK cells, neutrophils, and components of the MPS. CSS2 tumors, by contrast, demonstrated increased stromal cell scores, particularly fibroblasts and endothelial cells. CSS3 tumors displayed the highest scores for tumor epithelial cells, whereas CSS4 tumors exhibited the highest scores for plasma cells and the lowest scores for stromal cells (Figure 7G).

## 3 Discussion

**FIGURE 8:**
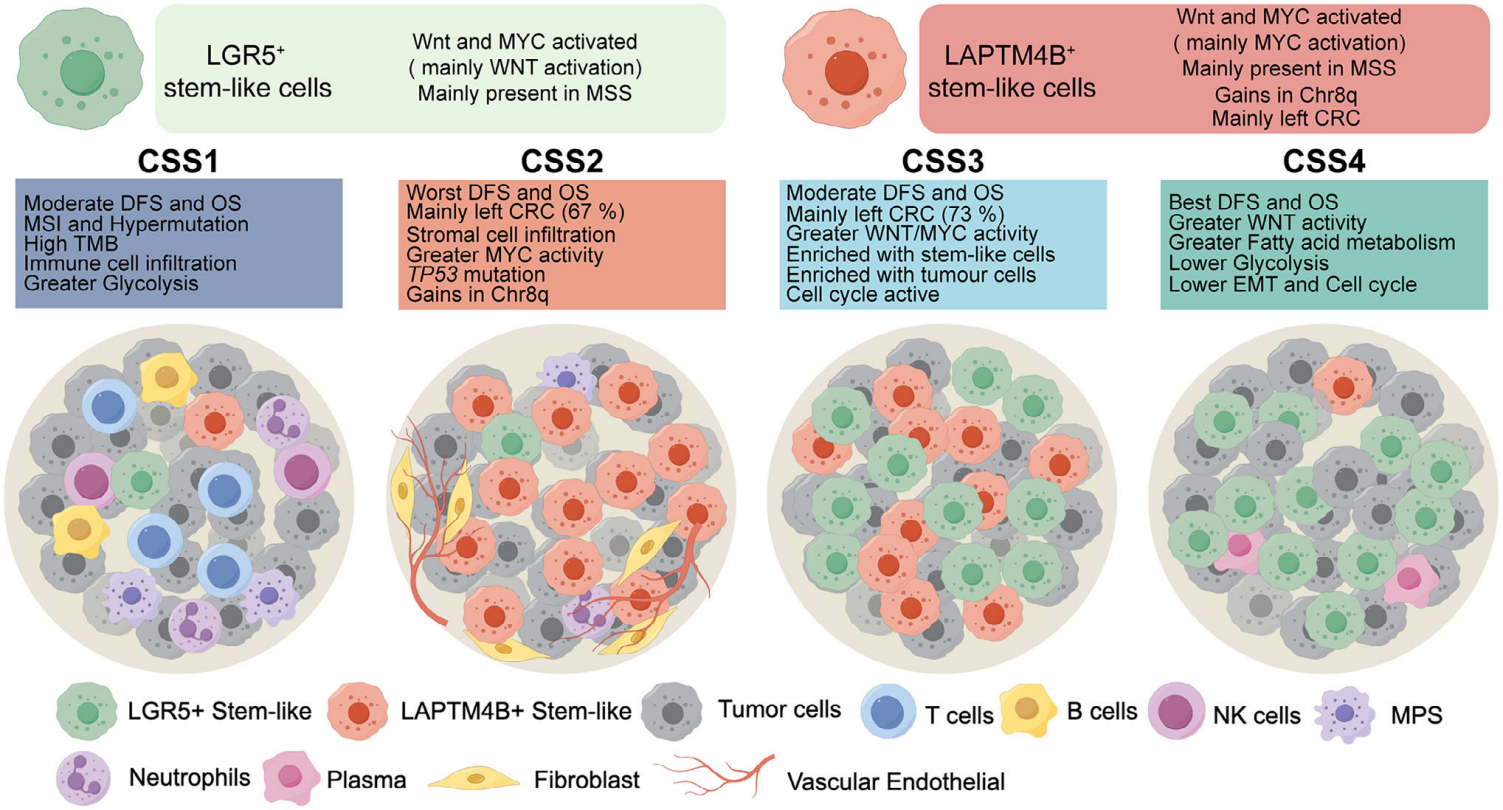
Stratification of CRC based on two tumor stem‐like cell subsets. The top panel summarizes the defining characteristics and marker genes of two tumor stem‐like cell subsets in CRC. The bottom panel delineates CRC stratification into CSS1, CSS2, CSS3, and CSS4 subtypes based on the relative abundance of these two stem‐like cell subsets, along with their associated clinical and molecular features.

Cancer stem‐like cells (CSCs) represent a pivotal subpopulation in CRC. However, their eradication remains challenging due to intrinsic heterogeneity and plasticity [19]. Advances in scRNA‐seq have significantly expanded insights into CRC's cellular landscape [11, 20]; however, the biological heterogeneity of CSCs remains incompletely characterized. This study integrates a multiomics framework—scRNA‐seq, bulk RNA‐seq, WES, and spatial transcriptomics—to dissect malignant cell heterogeneity in CRC. Beyond the canonical LGR5^+^ stem‐like cells, a novel subset characterized by LAPTM4B expression was identified. This distinct population exhibits aberrant 8q gain and *MYC* pathway hyperactivation. The intersection of LGR5^+^ and LAPTM4B^+^ stem‐like cells provide a basis for CRC stratification, leading to the CSS classification, which delineates four tumor subtypes with distinct biological and clinical attributes (Figure 8).

CRC recurrence is primarily driven by the persistence of CSCs, as current therapeutic strategies fail to eliminate this dynamic subpopulation. CSCs are not static entities but are modulated by diverse extrinsic and intrinsic factors [21]. Among CSC markers, LGR5 (GPR49) is the most extensively studied, with its elevated expression closely linked to WNT pathway activation. As a WNT target, LGR5 functions as an R‐spondin receptor to potentiate WNT signaling [22], a critical axis for CSC self‐renewal of stemness maintenance [23]. However, targeting LGR5^+^ CSCs as a therapeutic strategy remains contentious, as LGR5 ablation does not significantly impair tumor growth [24]. Moreover, LGR5 expression often declines during CRC progression, and higher LGR5 levels correlate with improved patient prognosis [25, 26, 27]. Emerging evidence suggests a potential tumor‐suppressive role of LGR5, possibly through its capacity to constrain cell proliferation [28, 29].

*LAPTM4B*, an oncogene closely linked to lysosomal function, was first identified in hepatocellular carcinoma (HCC), where its overexpression correlated with poor prognosis and enhances stemness [30, 31]. Beyond HCC, *LAPTM4B* has been implicated in tumor cell invasion and metastasis across multiple malignancies, including lung cancer [32], clear cell renal carcinoma [33], and osteosarcoma [34]. However, its association with cancer stem‐like cells in CRC remains unexplored. Leveraging a large‐scale CRC single‐cell atlas, this study identifies a distinct LAPTM4B^+^ stem‐like population strongly linked to CRC progression. Sparse LAPTM4B^+^ populations were also present in healthy intestinal tissues, and transcriptional similarities between rare Tuft cells and tumor stem‐like subsets were identified. Tuft cells exhibited residual LAPTM4B expression. Recent studies did identify the Tuft cells as regenerative stem cell‐like mediators in the intestinal epithelium [35]. However, whether LAPTM4B^+^ cells function parallel to LGR5^+^ cells in regulating physiological stem cell plasticity requires further validation through lineage tracing modeling.

*MYC*, a well‐characterized oncogene, plays a central role in tumor cell proliferation by preventing cell cycle arrest [36] and serves as a critical stemness regulator [37], maintaining self‐renewal capacity [38, 39]. MYC inhibition can induce tumor dormancy and promote a pluripotent‐like state [40]. In CRC, both WNT and MYC signaling pathways are essential for CSC maintenance, with their interplay supporting CSC survival and tumor progression [11, 41]. Previous studies have predominantly focused on LGR5^+^ cells characterized by active WNT signaling. This study confirms WNT and MYC activity in stem‐like cells, delineating them into two major subgroups: LGR5^+^ and LAPTM4B^+^. The activation of distinct signaling pathways may confer unique biological properties to CSCs, contributing to intratumoral heterogeneity in CRC. Further investigation is warranted to elucidate the interplay between these regulatory networks.

Genomic alterations in CSCs drive functional gene modifications, influencing tumor recurrence and chemoresistance [42]. This study identified a significant gain in the 8q region within LAPTM4B^+^ stem‐like cells, a finding validated using genomic data from 1066 patients. Notably, both LAPTM4B and MYC reside on chromosome 8q, a locus previously shown to exhibit higher DNA copy numbers in metastatic CRC than in primary tumors [43]. However, the functional consequences of 8q gain on CSC biology remain unclear, warranting further investigation. Additionally, *TP53* mutations are detected in approximately 40% of CRC cases [44], yet in CSS2 tumors, characterized by a predominance of LAPTM4B^+^ stem‐like cells, the mutation frequency escalates to 75%. Hilla et al. reported that *TP53* mutations enhance oncogenic potential and confer stem‐like characteristics in CRC cells [45], a phenomenon also observed in breast cancer [46] and osteosarcoma [47], underscoring the regulatory role of *TP53* in CSC biology. Given its pivotal role in tumorigenesis, further research is needed to delineate the interplay between *TP53* and LAPTM4B^+^ stem‐like cells.

This study further reveals that LGR5^+^ and LAPTM4B^+^ stem‐like cells exhibit opposing prognostic trends in CRC progression. A simple method leveraging the relative expression of LAPTM4B and LGR5 within tumor tissues may provide an accurate prediction of disease progression. However, certain limitations must be acknowledged, including a relatively short 3‐year follow‐up period and the retrospective study design, necessitating validation through prospective clinical investigations.

In conclusion, integrative analysis of scRNA‐seq, bulk RNA‐seq, WES, and spatial transcriptomics, identified a distinct LAPTM4B^+^ stem‐like population, predominantly present in MSS, *POLE* wild‐type, and left‐sided tumors, characterized by MYC pathway activation and 8q gain. LAPTM4B^+^ stem‐like cells are closely linked to CRC progression, contributing to tumor stratification through their interplay with LGR5^+^ stem‐like cells. These findings highlight the heterogeneity of CRC stem‐like cells, providing a refined framework for tumor classification and potential therapeutic targeting.

## 4 Materials and Methods

### 4.1 Patient and Tissue Sample Collection

Fresh tissue samples were collected from 148 patients with CRC undergoing surgical resection at the First Affiliated Hospital of Nanchang University. The specimens included tumor tissues, adjacent normal tissues, and peripheral blood mononuclear cells (PBMCs). Bulk RNA‐seq was performed on tumor and adjacent normal tissues, while WES was conducted on the tumor tissues and PBMCs. Additionally, fresh tumor tissues from 27 patients were subjected to scRNA‐seq to investigate intratumoral heterogeneity. Tumor staging and MSI status were assessed by pathologists, and clinical data were retrieved from medical records. The study was approved by the Ethics Committee of the First Affiliated Hospital of Nanchang University (2022‐CDYFYYLK‐06‐012), and written informed consent was obtained from all participants.

### 4.2 FAHNU‐SC Cohort: Sample Preparation and Processing for scRNA‐seq

Surgical resection samples from FAHNU‐SC were initially rinsed in cold RPMI 1640 medium, then preserved and transported in MACS Tissue Storage Solution (Cat No. 130‐100‐008, Miltenyi). The tissues were finely minced using sterile surgical blades and enzymatically dissociated with the Tumor Dissociation Kit (Cat No. 130‐095‐929, Miltenyi) following the manufacturer's protocol, followed by DNase treatment. Red blood cells were eliminated using red blood cell lysis solution (Cat No. 130‐094‐183, Miltenyi). The viability and concentration of live cells were assessed using a Countstar Rigel S2 cell analyzer. Debris and dead cells were removed using corresponding purification kits (Cat. Nos. 130‐109‐398/130‐090‐101, Miltenyi), and samples were centrifuged at room temperature before resuspension in phosphate‐buffered saline (PBS) containing 0.04% bovine serum albumin (BSA). scRNA‐seq libraries were prepared using the SeekOne‐Digital Droplet platform and sequenced on an Illumina NovaSeq 6000 platform (PE150). Raw reads underwent quality control and primer sequence trimming using fastp [48]. The processed reads were aligned to the human reference genome (GRCh38/hg38) via the SeekOne Tools pipeline, generating transcript expression matrices. The expression matrix was analyzed using scanpy (version 1.9.3) [49], with Scrublet (version 0.2.3) [50] applied to remove doublet cells. Cells with fewer than 200 transcripts or with mitochondrial gene content exceeding 40% were excluded from downstream analyses.

### 4.3 Single‐Cell RNA Sequencing Data Analysis

In this study, scRNA‐seq data from the FAHNU‐SC cohort were integrated with publicly available scRNA‐seq datasets, including paired tumor tissues (*n* = 152) and normal adjacent tissues (*n* = 73). The public datasets had been preannotated with coarse cell types, and mitochondrial genes were excluded from all datasets prior to further analysis. The combined dataset was normalized and standardized using the Scanpy toolkit. Subsequently, the scArches (version 0.5.0) [51] tool was employed to integrate all single‐cell datasets, following strategies outlined in previous studies [52]. In brief, the annotated public data served as a reference, and the CellTypist (version 1.6.2) [53] logistic regression classifier was used to analyze and annotate each dataset separately by source. Batch alignment was then performed based on data source and donor information.

The consensus non‐negative matrix factorization (cNMF) algorithm, incorporating batch variables, was applied to identify latent features consisting of coexpressed genes within tumor epithelial cells (version 1.5) [54]. Large‐scale chromosomal CNVs for each cell were computed using the inferCNV tool, implemented in both R (version 1.3.3) and Python (version 0.4.5). Genomic instability was assessed using the genomicInstability R package (version 1.10.0), which estimates the relationship between gene expression and genomic location. The aREA algorithm was employed to quantify the enrichment of consecutive gene sets (locus blocks) within gene expression profiles, thereby estimating GIS for each analyzed cell. For single‐cell data, AUCell (version 1.26.0) [55] scores were calculated to assess the activity of different gene sets, and the CytoTRACE2 (version 1.0.0) [56]. R package was used to predict the relative differentiation states of cells. RNA velocity estimated using the scTour Python package (version 1.0.0) [57], and metabolic fluxes in different cells were inferred using the scFEA (version 1.1.2) [58].

Following prior research methodologies [59], the distribution preference of each cell subpopulation across different groups was quantified by calculating the ratio of observed to expected cell numbers (Ro/e). Ro/e > 1 indicates enrichment exceeding stochastic expectation in tissue compartments, while values < 1 denote depletion relative to expectation. Intercellular communication was assessed using the CellChat tool (version 2.1.0) [60]. Data visualization was performed using the OmicVerse toolkit (version 1.6.4) [61].

### 4.4 Whole‐Exome Sequencing Data Analysis

Raw sequencing data were processed initially using fastp (version 0.23.2) [48] to trim adapter sequences and remove low‐quality reads. Following filtering, clean reads were aligned to the GRCh38/hg38 reference genome using the Burrows‐Wheeler Aligner (BWA, version 0.7.17) [62]. The resulting alignment files were further processed using a suite of tools, including the Genome Analysis Toolkit 4 (GATK4, version 4.2.6.1), SAMtools (version 1.13) [63], and Picard, to generate and refine the BAM files. For somatic variant detection, the corresponding BAM files from the patients' PBMCs were used as controls. Somatic single nucleotide variants (SNVs) and insertions/deletions (INDELs) were identified following the best practices outlined in GATK4‐Mutect2 [64, 65]. These variants were then filtered and annotated using the Variant Effect Predictor (VEP, version 106) [66]. Somatic CNVs were inferred using Sequenza (version 3.0.0) [67]. The tumor mutational burden (TMB) was calculated as the total number of nonsilent somatic mutations. To visualize the mutation landscape across different genes, a waterfall plot was generated using the ComplexHeatmap package (version 2.14.0).

### 4.5 Bulk Transcriptome Data Analysis

Bulk transcriptome data, obtained from both cancerous and adjacent normal tissues, underwent initial quality control with fastp [48]. Clean reads were aligned to the GRCh38/hg38 reference genome using the STAR aligner (version 2.7.2b) [68]. Gene‐level quantification was then performed using Salmon (version 1.9.0) [69]. To assess the functional implications of gene expression, Gene Set Variation Analysis (GSVA) was carried out using gene sets curated from MSigDB. For integrative analysis across five distinct cohorts, batch effects were corrected using the ComBat algorithm (version 3.5.4) [70], and all gene expression values were log‐transformed, centered to zero, and unit variance‐scaled for visualization. This normalization minimized the impact of cohort‐specific sequencing biases. Using the CRC single‐cell atlas as a reference, the linear support vector regression (SVR) algorithm in CIBERSORT was employed to estimate the proportion of each cell type in the bulk transcriptome data, performed using the IOBR package (version 0.99.9) [71]. LGR5^+^/LAPTM4B^+^ stem‐like cell signatures were defined through following approach: (1) filtering significantly upregulated genes in Epi T cells (Epi T cells vs. other cells), (2) comparing intraepithelial subpopulation comparisons (LGR5^+^/LAPTM4B^+^ vs. other Epi T cell subpopulations), and (3) selecting intersectional gene sets. LGR5^+^/LAPTM4B^+^ stem‐like cell signatures for each sample were calculated based on the median expression levels of the corresponding gene sets. CRC samples were stratified into four subtypes based on the LGR5^+^ and LAPTM4B^+^ signatures, with the median expression level serving as the threshold due to the skewed data distribution. Additionally, the deep learning‐based CRC CMS classification model, DeepCC (version 0.1.1) [72], was used to identify CMS based on the gene expression profiles of the CRC samples. Samples with complete documentation of both OS and DFS, and nonzero survival times for both endpoints, were retained for survival analysis following filtration (*n* = 985).

### 4.6 Statistical Analysis and Visualization

The D'Agostino–Pearson test was performed to assess the normality of data distributions. For datasets deviating from normality, nonparametric tests were applied. Based on the distribution, either parametric or nonparametric *t*‐tests compared differences between two groups. Pearson correlation analysis evaluated the linear relationship between continuous variables. Fisher's exact test analyzed differences in categorical variables. The false discovery rate (FDR) was controlled using the Benjamini–Hochberg (BH) correction. Kaplan–Meier survival analysis and visualization were performed using the survminer R package (version 0.4.9), with log‐rank tests comparing survival curves to identify significant differences between groups. Multivariable Cox proportional hazards regression identified independent prognostic factors associated with survival. All statistical analyses and visualizations were carried out using R (version 4.2.3) or GraphPad Prism 9.5, and flowcharts were created with the Figdraw tool. A *p* value of less than 0.05 was considered statistically significant.

## Author Contributions

Yangyang Fang, Kuai Yu, and Aiping Le conceptualized, designed, and supervised the study. Yangyang Fang collected and visualized the data. Yangyang Fang, Tianmei Fu, and Ziqing Xiong conducted the related wet‐lab experiments. Yangyang Fang and Tianmei Fu performed the statistical analysis and interpretation of all data. Yangyang Fang and Tianmei Fu prepared the manuscript, which was reviewed by Qian Zhang, Wei Liu, and Aiping Le. All the authors approved the final version of the manuscript. All the listed authors meet the criteria for authorship, and no one who meets these criteria has been omitted. Aiping Le is the guarantor of this study. All the authors have read and approved the final manuscript.

## Ethics Statement

Clinical approval: This study was approved by the Ethics Committee of the First Affiliated Hospital of Nanchang University (2022‐CDYFYYLK‐06‐012). Informed consent was obtained from all participants prior to their inclusion in the research. Animal studies: Not applicable.

## Conflicts of Interest

The authors declare no conflicts of interest.

## Supporting information




**Supporting File 1**: mco270284‐sup‐0001‐SuppMat.docx.

## Data Availability

The scRNA‐seq, bulk RNA‐seq, and WES data generated in this study have been deposited in the Genome Sequence Archive (GSA) database under the accession number HRA004701. Processed public CRC scRNA‐seq datasets, including SG, KUL, and SMC, are accessible via the Synapse platform under accession code syn26844071. The Board dataset is available for download from the Gene Expression Omnibus (GEO) at https://www.ncbi.nlm.nih.gov/geo/ under accession number GSE178341. The transcriptomic and genomic Level 3 data analyzed in this study were obtained from The Cancer Genome Atlas (TCGA) database and downloaded in March 2023. The transcriptomic data for the SG‐BULK cohort were retrieved from Synapse under accession code syn26720761, and the genomic data for this cohort were downloaded from the European Genome–phenome Archive (EGA) under accession number EGAD00001008543 (https://ega‐archive.org/datasets/EGAD00001008543). Data for the AC‐ICAM cohort were acquired from cBioPortal (www.cbioportal.org). Additionally, data for the MCC cohort were obtained from GEO under accession number GSE17536. High‐resolution spatial transcriptomic data for CRC were downloaded from https://www.10xgenomics.com/products/visium‐hd‐spatial‐gene‐expression/dataset‐human‐crc. We acknowledge the support provided by these databases and express our gratitude to the contributors who uploaded these valuable datasets.
