# Supplementary material for: Novel Lysosomal‐Associated Transmembrane Protein 4B‐Positive Stem‐Like Cell Subpopulation Characterizes High‐Risk Colorectal Cancer Subtypes
Source: MedComm (2020). 2025 Jul 13;6(7):e70284. doi: 10.1002/mco2.70284 (PMC12256684; doi:10.1002/mco2.70284)
Supplement: Supplementary file 1 — Supporting File 1: mco270284‐sup‐0001‐SuppMat.docx. [file MCO2-6-e70284-s001.docx]

**Novel Lysosomal-associated Transmembrane Protein 4B-positive Stem-Like Cell Subpopulation Characterizes High-risk Colorectal Cancer Subtypes**

Yangyang Fang^1#^, Tianmei Fu^1#^, Ziqing Xiong^1^, Qian Zhang^1^, Wei Liu^1^, Kuai Yu^1*^, Aiping Le^1*^

^1^ Department of Transfusion Medicine, Key Laboratory of Jiangxi Province for Transfusion Medicine, The First Affiliated Hospital, Jiangxi Medical College, Nanchang University.

^#^These authors contributed equally to this work.

^*^Correspondence:

[yukuai1949@foxmail.com](mailto:yukuai1949@foxmail.com) (Kuai Yu)

ndyfy00973@ncu.edu.cn (Aiping Le)


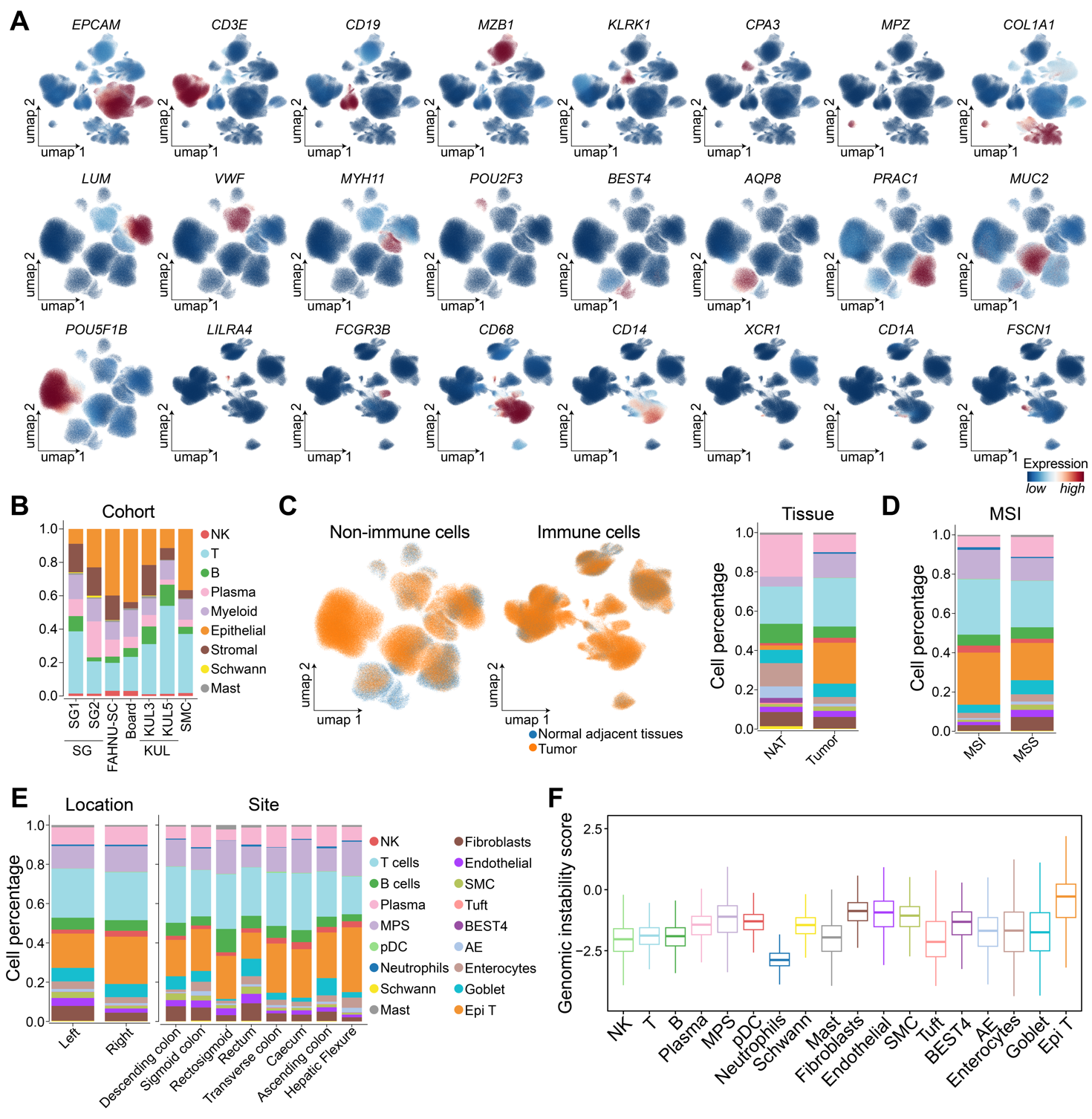


**Figure S1 The establishment of the CRC single-cell atlas.** (A) UMAP plot depicting the marker genes across different cell types. (B) A bar chart illustrating the proportions of various cell types across different cohorts. (C) UMAP plot (left) showing samples from human colorectal cancer tissue and normal adjacent tissue, accompanied by a bar chart (right) representing the corresponding cell type proportions. (D) Bar chart displaying the proportions of different cell types in MSI and MSS tumor tissues. (E) Bar chart indicating the proportions of different cell types in left-sided and right-sided colorectal cancer. (F) Box plot demonstrating the genomic instability scores across all cellular subpopulations within colorectal cancer.

**
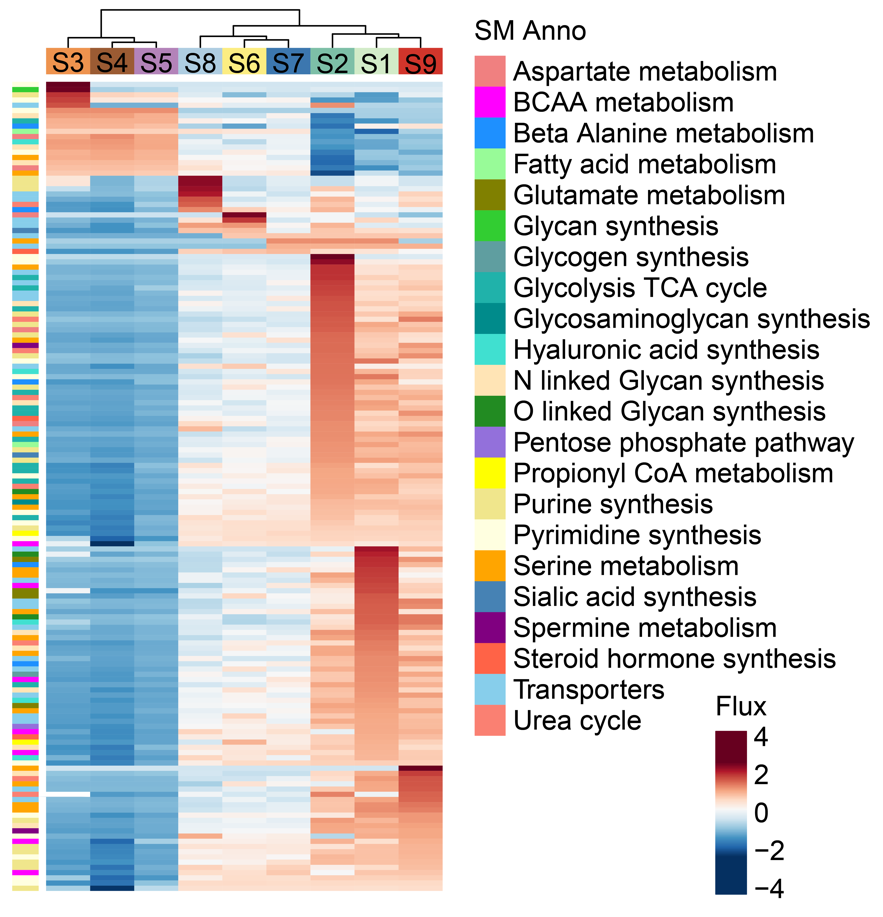
**

**Figure S2** Heatmap presents the predicted metabolic signatures for various Epi T cell subsets, derived from inferred metabolic flux data.

**
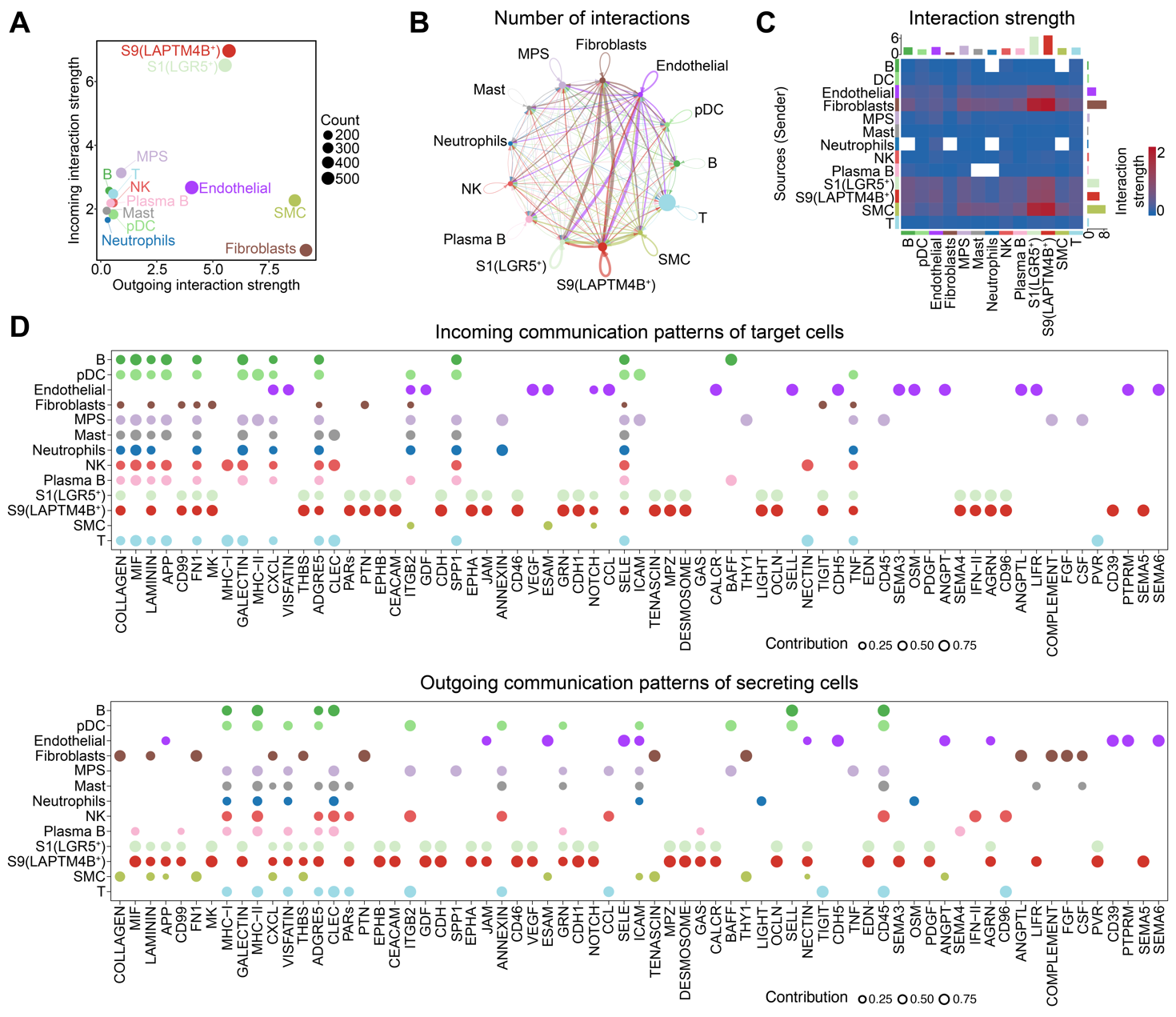
**

**Figure S3 Cell communication visualization.** (A) The dot plots show the strength of the interactions of the different cell types. (B) Circle plot delineates the number of interactions among 13 cell types in CRC. (C) The heatmap delineates the strength of interactions associated with intercellular interactions among distinct cell types. (D) The bubble plot delineates the incoming and outgoing communication patterns between target cell populations and secretory cell clusters.

**
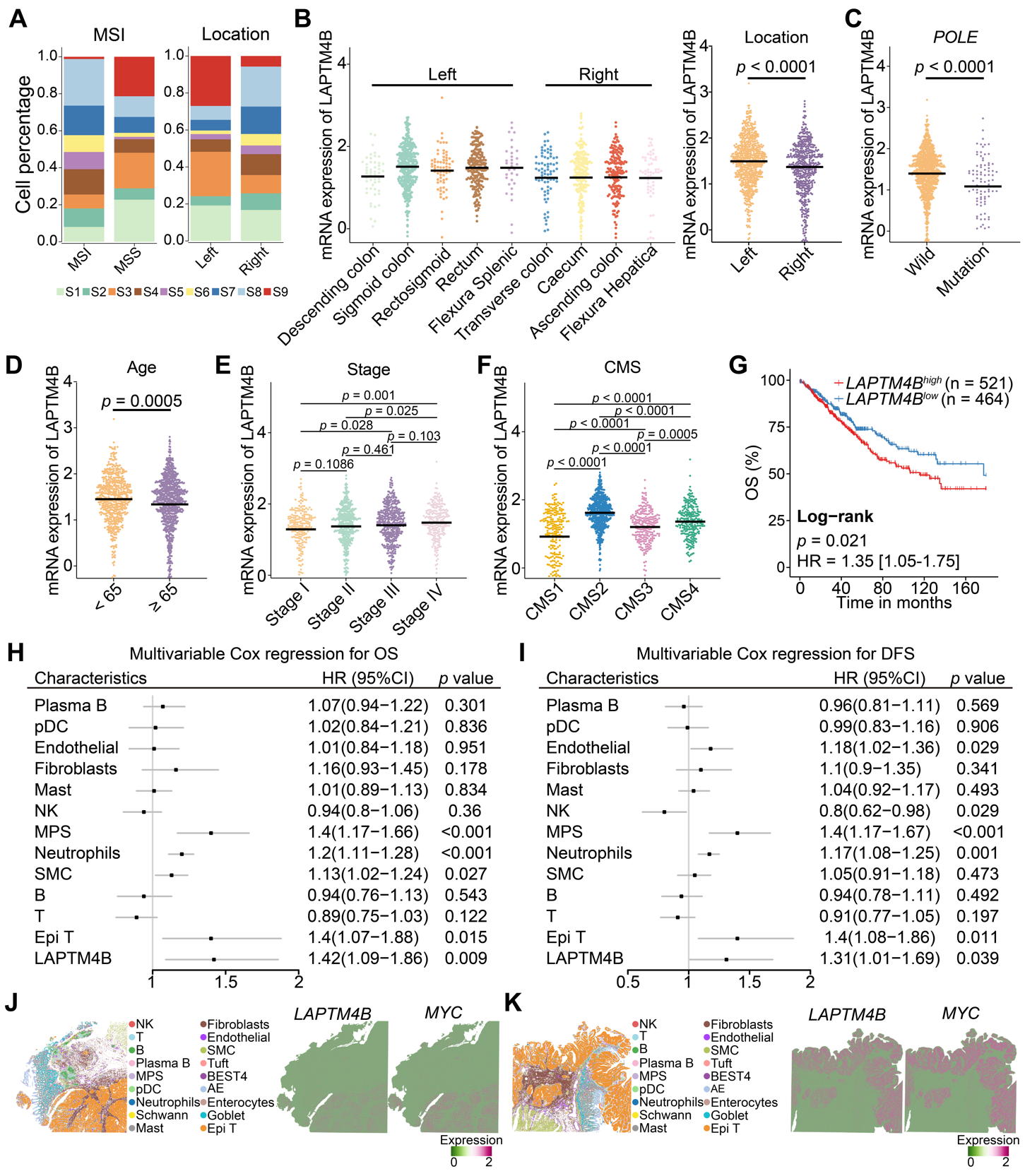
**

**Figure S4 LAPTM4B is identified as a marker gene for the S9 cell subsets.** (A) Bar charts display the proportions of S1-S9 subclusters in both MSI and MSS tumors (left), and in left-sided versus right-sided tumors (right). (B) The mRNA expression levels of *LAPTM4B* are shown according to tumor location, categorized into left-sided and right-sided tumors (C-F) *LAPTM4B* mRNA expression levels across tumors with different *POLE* mutation statuses (C), age groups (D), pathological stages (E), and CMS classifications (F). (G) Kaplan-Meier survival analysis for OS based on *LAPTM4B* expression, analyzed using the Log-rank test (n = 985). (H-I) Forest plots summarizing the impact of *LAPTM4B* on OS (H) and DFS (I) were generated using multivariable Cox regression, adjusted for infiltration levels of various cell populations as covariates. (J-K) Expression of *LAPTM4B* and *MYC* in two additional CRC spatial transcriptomic samples. *P*-values for comparisons between two groups were calculated using two-sided t-tests.


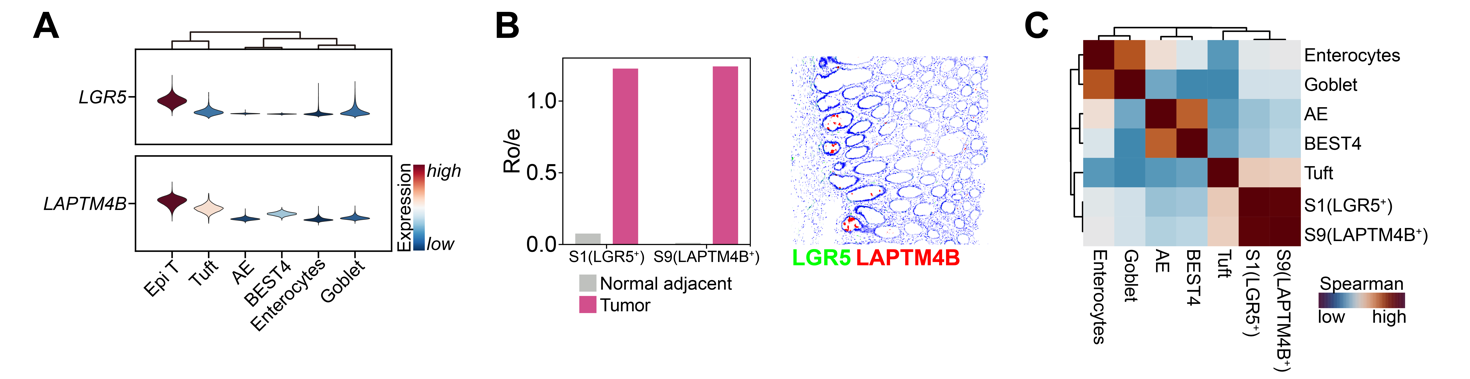


**Figure S5** **Distribution of two distinct stem-like cell populations within normal intestinal tissue.** (A) The violin plot illustrates the expression of the S1 and S9 subgroup marker genes (*LGR5* and *LAPTM4B*) in non-malignant epithelial cells. (B) The bar chart illustrates the distributional preferences of the S1 and S9 cell subpopulations between normal and tumor tissues (left). Representative mIHC images of healthy intestinal tissue (right). (C) The heatmap depicts the correlative relationships between the expression profiles of two tumor stem-like cell populations and non-malignant epithelial cells, with red color-coding indicating positive correlations and blue representing negative correlations.

**
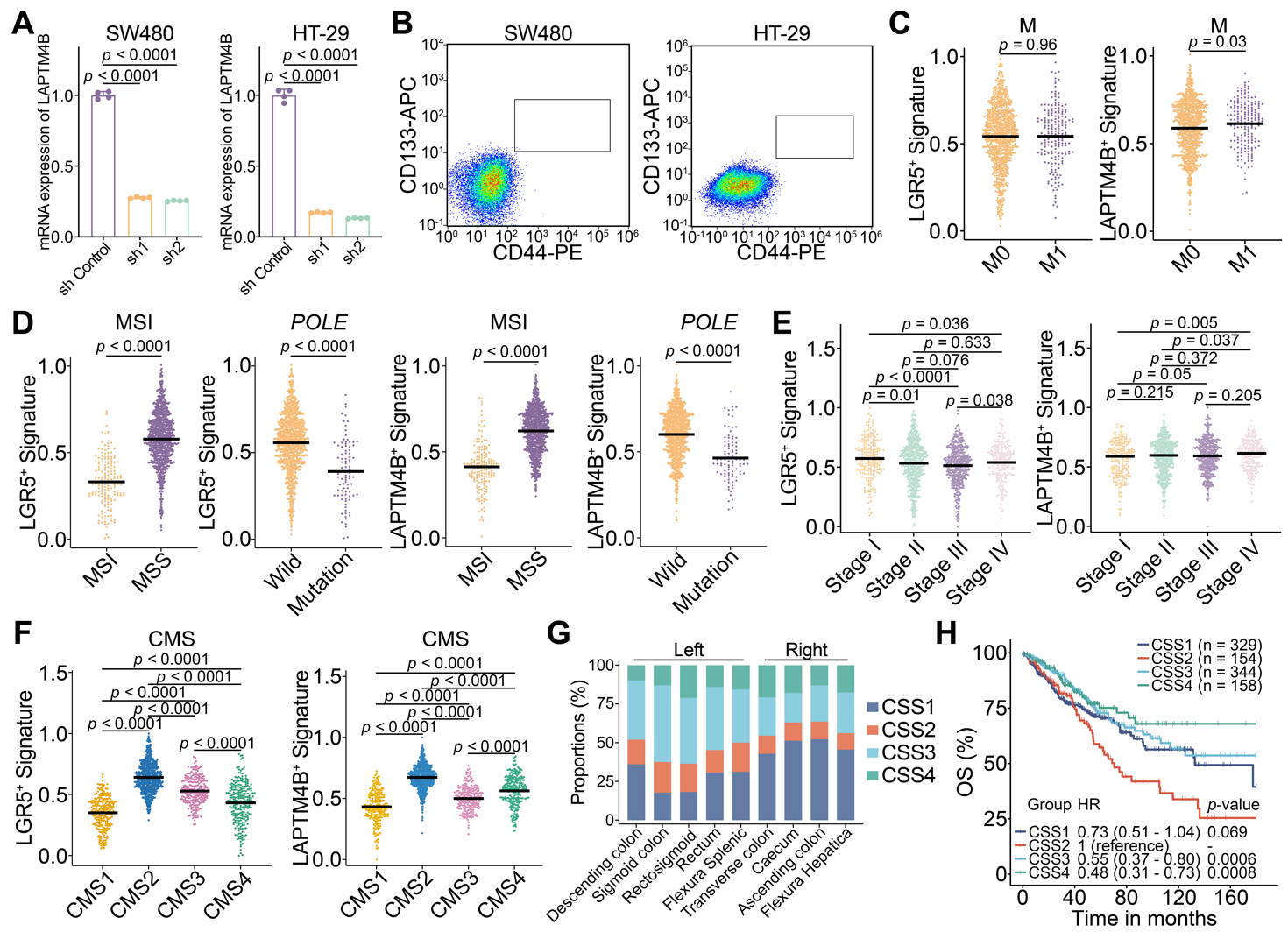
**

**Figure S6 LGR5^+^ and LAPTM4B^+^ stem cells jointly define CRC stratification.** (A) mRNA levels of *LAPTM4B* in knockdown CRC cells compared to their negative controls. (B) Representative flow cytometry plots showing PE and APC fluorescence channels for blank controls. (C) Differences in LGR5^+^ and LAPTM4B^+^ signatures between tumors with distant metastasis and those without. (D) Comparison of LGR5^+^ and LAPTM4B^+^ signatures among MSI and MSS tumors, as well as between *POLE* wild-type and *POLE* mutant tumors. (E) Variations in LGR5^+^ and LAPTM4B^+^ signatures across different pathological stages of tumors. (F) Differences in LGR5^+^ and LAPTM4B^+^ signatures among tumors classified by different CMS subtypes. (G) Bar graph illustrating the distribution of different CSS subtypes across various tumor locations, divided into left and right sides. (H) OS survival curves for different CSS subtypes (n = 985). *P*-values for pairwise comparisons were calculated using a two-tailed t-test. Significance in survival analysis was determined by the log-rank test.

**
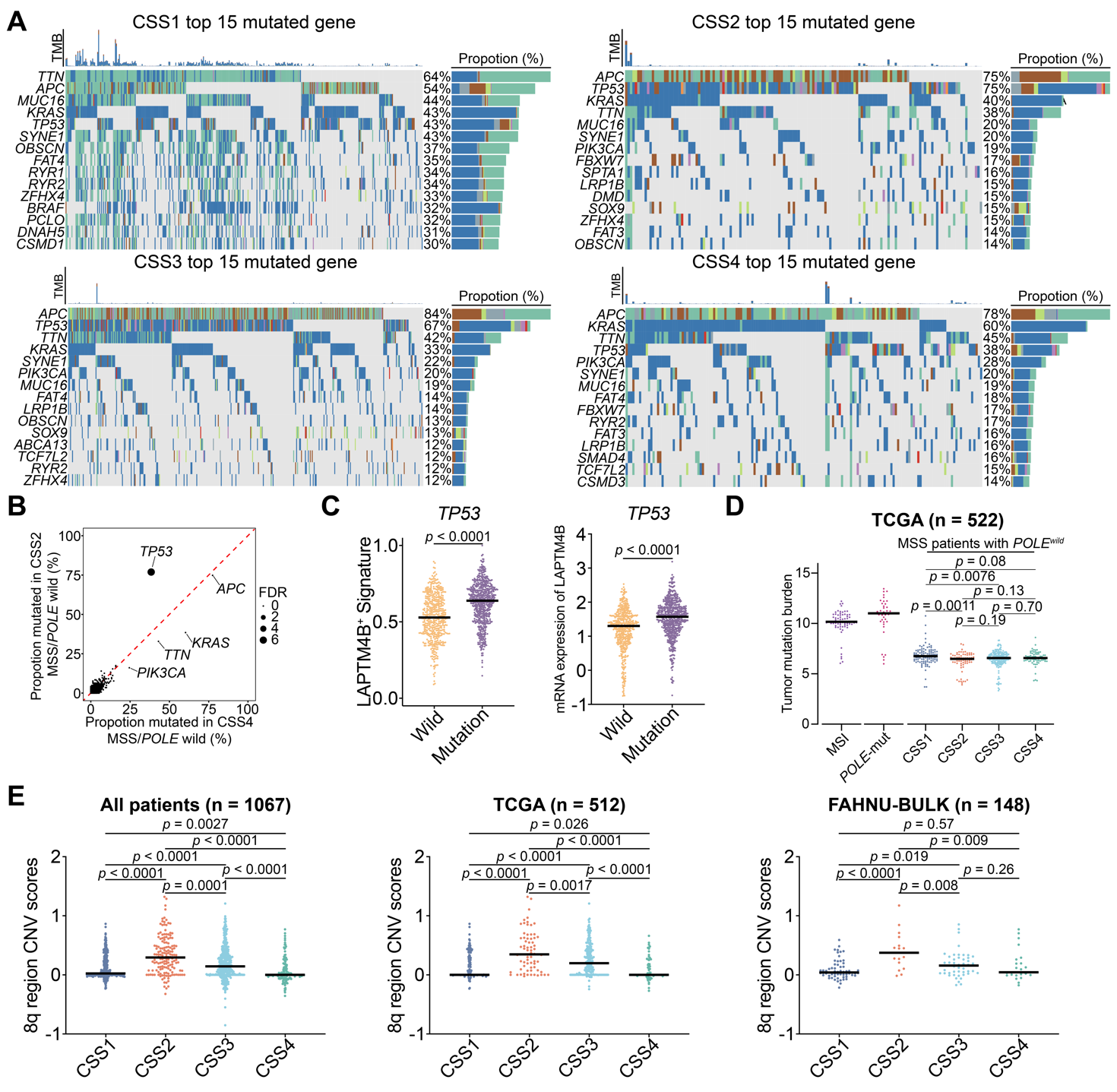
**

**Figure S7 Genomic characteristics of CRC stem-like cell subtypes.** (A) Waterfall plot displaying the top 15 mutated genes ranked by mutation frequency across the four CRC stem-like cell subtypes. (B) Scatter plot illustrating gene mutation frequencies in MSS and *POLE* wild-type tumors, comparing the CSS2 and CSS4 subtypes. The size of the dots corresponds to the Q values, adjusted by BH correction, from two-sided Fisher's exact tests. (C) Comparison of LAPTM4B^+^ stem-like cell signature scores (left) and *LAPTM4B* mRNA levels (right) between *TP53*-mutant and *TP53* wild-type tumors. (D) Comparison of tumor mutational burden across distinct CSS subtypes within the TCGA cohort. (E) CNV scores in the chromosome 8q region across samples with different CSS subtypes. All *p*-values were determined by two-sided t-tests.

**Table S1.** Clinical data from 152 primary CRC patients utilized to construct the single-cell atlas

| **Patient.ID** | **Dataset** | **Gender** | **Age** | **MSI** | **Location** | **Stage** | **NAT** |
| --- | --- | --- | --- | --- | --- | --- | --- |
| C103 | Board | Male | 45 | MSS | Sigmoid colon | II | FALSE |
| C104 | Board | Male | 81 | MSS | Transverse colon | III | FALSE |
| C105 | Board | Male | 71 | MSS | Ascending colon | IV | FALSE |
| C106 | Board | Male | 67 | MSI | Caecum | IV | TRUE |
| C107 | Board | Male | 62 | MSS | Transverse colon | III | TRUE |
| C109 | Board | Female | 60 | MSI | Ascending colon | III | TRUE |
| C110 | Board | Male | 59 | MSI | Hepatic Flexure | III | TRUE |
| C111 | Board | Female | 62 | MSI | Hepatic Flexure | III | TRUE |
| C112 | Board | Male | 50 | MSS | Caecum | III | TRUE |
| C113 | Board | Male | 52 | MSS | Ascending colon | II | TRUE |
| C114 | Board | Male | 76 | MSI | Transverse colon | III | TRUE |
| C115 | Board | Male | 72 | MSI | Ascending colon | III | TRUE |
| C116 | Board | Male | 72 | MSI | Ascending colon | III | TRUE |
| C118 | Board | Male | 77 | MSI | Ascending colon | II | FALSE |
| C119 | Board | Male | 48 | MSI | Ascending colon | III | TRUE |
| C122 | Board | Female | 82 | MSI | Hepatic Flexure | III | TRUE |
| C123 | Board | Male | 58 | MSI | Ascending colon | III | TRUE |
| C124 | Board | Female | 81 | MSS | Rectosigmoid | IV | TRUE |
| C125 | Board | Male | 49 | MSS | Sigmoid colon | III | TRUE |
| C126 | Board | Female | 81 | MSS | Caecum | IV | TRUE |
| C129 | Board | Female | 35 | MSS | Descending colon | III | TRUE |
| C130 | Board | Female | 89 | MSI | Caecum | IV | TRUE |
| C132 | Board | Male | 42 | MSI | Descending colon | IV | TRUE |
| C133 | Board | Female | 73 | MSS | Ascending colon | IV | TRUE |
| C134 | Board | Male | 58 | MSS | Sigmoid colon | IV | TRUE |
| C135 | Board | Male | 90 | MSS | Caecum | I | TRUE |
| C136 | Board | Male | 60 | MSS | Rectosigmoid | III | TRUE |
| C137 | Board | Female | 56 | MSI | Ascending colon | III | TRUE |
| C138 | Board | Male | 60 | MSI | Transverse colon | III | TRUE |
| C139 | Board | Female | 61 | MSI | Ascending colon | IV | TRUE |
| C140 | Board | Male | 35 | MSS | Sigmoid colon | I | TRUE |
| C142 | Board | Female | 51 | MSI | Hepatic Flexure | III | TRUE |
| C143 | Board | Female | 55 | MSI | Caecum | III | TRUE |
| C144 | Board | Male | 91 | MSI | Caecum | III | FALSE |
| C145 | Board | Female | 59 | MSS | Ascending colon | IV | FALSE |
| C146 | Board | Female | 64 | MSI | Ascending colon | III | FALSE |
| C147 | Board | Female | 73 | MSI | Caecum | IV | FALSE |
| C149 | Board | Female | 47 | MSS | Ascending colon | IV | FALSE |
| C150 | Board | Male | 70 | MSS | Rectosigmoid | III | FALSE |
| C151 | Board | Female | 76 | MSI | Caecum | III | TRUE |
| C152 | Board | Female | 77 | MSI | Hepatic Flexure | II | TRUE |
| C153 | Board | Female | 36 | MSS | Sigmoid colon | II | FALSE |
| C154 | Board | Female | 78 | MSI | Transverse colon | II | FALSE |
| C155 | Board | Female | 53 | MSS | Ascending colon | III | TRUE |
| C156 | Board | Female | 76 | MSI | Ascending colon | III | FALSE |
| C157 | Board | Female | 62 | MSS | Descending colon | IV | TRUE |
| C158 | Board | Female | 84 | MSI | Ascending colon | III | FALSE |
| C159 | Board | Male | 57 | MSS | Caecum | III | FALSE |
| C160 | Board | Female | 52 | MSS | Ascending colon | IV | FALSE |
| C161 | Board | Male | 71 | MSS | Ascending colon | III | FALSE |
| C162 | Board | Male | 47 | MSS | Ascending colon | IV | TRUE |
| C163 | Board | Female | 71 | MSI | Transverse colon | IV | FALSE |
| C164 | Board | Female | 60 | MSI | Ascending colon | IV | FALSE |
| C165 | Board | Male | 69 | MSI | Ascending colon | IV | TRUE |
| C166 | Board | Male | 60 | MSS | Sigmoid colon | III | FALSE |
| C167 | Board | Female | 72 | MSI | Caecum | III | FALSE |
| C168 | Board | Male | 82 | MSI | Ascending colon | II | FALSE |
| C169 | Board | Male | 62 | MSI | Transverse colon | II | FALSE |
| C170 | Board | Female | 77 | MSI | Ascending colon | III | TRUE |
| C171 | Board | Male | 61 | MSS | Ascending colon | II | FALSE |
| C172 | Board | Female | 61 | MSS | Ascending colon | III | FALSE |
| C173 | Board | Female | 49 | MSI | Descending colon | IV | FALSE |
| CRC-JSC-S02 | SG-SG2 | Male | 47 | MSI | Caecum | III | FALSE |
| CRC-JSC-S03 | SG-SG2 | Female | 59 | MSI | Ascending colon | II | FALSE |
| CRC-JSC-S04 | SG-SG2 | Female | 65 | MSS | Sigmoid colon | III | FALSE |
| CRC-JSC-S05 | SG-SG2 | Male | 79 | MSI | Caecum | II | FALSE |
| CRC-JSC-S06 | SG-SG2 | Male | 80 | MSS | Rectum | III | FALSE |
| CRC-JSC-S07 | SG-SG2 | Male | 66 | MSS | Rectosigmoid | II | FALSE |
| CRC-JSC-S08 | SG-SG2 | Male | 64 | MSS | Rectum | III | FALSE |
| CRC-JSC-S10 | SG-SG2 | Male | 50 | MSS | Caecum | II | FALSE |
| CRC-JSC-S12 | SG-SG2 | Female | 79 | MSS | Sigmoid colon | III | FALSE |
| CRC-JSC-S13 | SG-SG2 | Male | 72 | MSS | Ascending colon | III | FALSE |
| CRC-JSC-S14 | SG-SG2 | Male | 66 | MSS | Rectum | III | FALSE |
| CRC-JSC-S15 | SG-SG2 | Male | 72 | MSS | Sigmoid colon | II | FALSE |
| CRC2783 | SG-SG1 | Female | 31 | MSI | Transverse colon | III | TRUE |
| CRC2786 | SG-SG1 | Male | 68 | MSS | Ascending colon | II | TRUE |
| CRC2787 | SG-SG1 | Female | 61 | MSS | Rectum | II | TRUE |
| CRC2794 | SG-SG1 | Female | 65 | MSS | Descending colon | II | TRUE |
| CRC2795 | SG-SG1 | Male | 58 | MSS | Rectum | III | TRUE |
| CRC2801 | SG-SG1 | Male | 50 | MSS | Descending colon | III | TRUE |
| CRC2803 | SG-SG1 | Male | 60 | MSS | Rectum | III | TRUE |
| CRC2810 | SG-SG1 | Female | 76 | MSS | Sigmoid colon | III | TRUE |
| CRC2811 | SG-SG1 | Male | 63 | MSS | Sigmoid colon | III | TRUE |
| CRC2816 | SG-SG1 | Male | 59 | MSS | Rectum | III | TRUE |
| CRC2817 | SG-SG1 | Male | 67 | MSI | Caecum | III | TRUE |
| CRC2821 | SG-SG1 | Female |  | MSS | Ascending colon | III | FALSE |
| CRC2841 | SG-SG1 | Male | 64 | MSS | Sigmoid colon | III | TRUE |
| CRC2899 | SG-SG1 | Female | 76 | MSS | Ascending colon | III | TRUE |
| SC001 | KUL-KUL3 | Female | 81 | MSI | Caecum | II | TRUE |
| SC019 | KUL-KUL3 | Female | 86 | MSS | Rectosigmoid | III | TRUE |
| SC021 | KUL-KUL3 | Female | 50 | MSS | Sigmoid colon | IV | TRUE |
| SC024 | KUL-KUL3 | Female | 33 | MSS | Sigmoid colon | II | TRUE |
| SC027 | KUL-KUL3 | Male | 81 | MSS | Sigmoid colon | I | TRUE |
| SC028 | KUL-KUL3 | Male | 52 | MSS | Sigmoid colon | II | TRUE |
| SC029 | KUL-KUL3 | Female | 77 | MSS | Caecum | III | TRUE |
| SC030 | KUL-KUL3 | Male | 84 | MSS | Ascending colon | II | TRUE |
| SC031 | KUL-KUL3 | Male | 85 | MSS | Sigmoid colon | I | TRUE |
| SC035 | KUL-KUL5 | Male | 44 | MSI | Ascending colon | II | TRUE |
| SC040 | KUL-KUL5 | Female | 68 | MSS | Ascending colon | II | TRUE |
| SC041 | KUL-KUL5 | Male | 77 | MSS | Rectosigmoid | III | TRUE |
| SC043 | KUL-KUL5 | Female | 55 | MSS | Sigmoid colon | III | TRUE |
| SC044 | KUL-KUL5 | Female | 80 | MSI | Caecum | III | TRUE |
| SMC01 | SMC | Female | 64 | MSS | Rectum | II | TRUE |
| SMC02 | SMC | Male | 66 | MSS | Rectum | III | TRUE |
| SMC03 | SMC | Female | 83 | MSI | Hepatic Flexure | III | TRUE |
| SMC04 | SMC | Male | 69 | MSS | Sigmoid colon | III | TRUE |
| SMC05 | SMC | Female | 58 | MSS | Ascending colon | II | TRUE |
| SMC06 | SMC | Male | 46 | MSI | Hepatic Flexure | III | TRUE |
| SMC07 | SMC | Female | 67 | MSS | Ascending colon | I | TRUE |
| SMC08 | SMC | Male | 68 | MSS | Sigmoid colon | III | TRUE |
| SMC09 | SMC | Male | 75 | MSS | Sigmoid colon | II | TRUE |
| SMC10 | SMC | Female | 77 | MSI | Ascending colon | II | TRUE |
| SMC11 | SMC | Female | 38 | MSS | Sigmoid colon | III | FALSE |
| SMC14 | SMC | Male | 77 | MSS | Rectosigmoid | III | FALSE |
| SMC15 | SMC | Male | 56 | MSS | Sigmoid colon | II | FALSE |
| SMC16 | SMC | Male | 59 | MSS | Ascending colon | III | FALSE |
| SMC17 | SMC | Male | 47 | MSS | Hepatic Flexure | III | FALSE |
| SMC18 | SMC | Female | 63 | MSS | Sigmoid colon | II | FALSE |
| SMC19 | SMC | Female | 80 | MSS | Ascending colon | III | FALSE |
| SMC20 | SMC | Female | 65 | MSS | Ascending colon | III | FALSE |
| SMC21 | SMC | Male | 51 | MSS | Rectum | IV | FALSE |
| SMC22 | SMC | Male | 76 | MSS | Sigmoid colon | III | FALSE |
| SMC23 | SMC | Female | 67 | MSS | Ascending colon | III | FALSE |
| SMC24 | SMC | Female | 48 | MSI | Ascending colon | I | FALSE |
| SMC25 | SMC | Female | 57 | MSS | Sigmoid colon | IV | FALSE |
| FAHNU-SC01 | FAHNU-SC | Male | 52 | MSI | Ascending colon | IV | FALSE |
| FAHNU-SC02 | FAHNU-SC | Male | 73 | MSS | Descending colon | II | FALSE |
| FAHNU-SC03 | FAHNU-SC | Male | 45 | MSS | Ascending colon | II | FALSE |
| FAHNU-SC04 | FAHNU-SC | Male | 67 | MSS | Rectum | II | FALSE |
| FAHNU-SC05 | FAHNU-SC | Male | 79 |  | Rectum | III | FALSE |
| FAHNU-SC06 | FAHNU-SC | Male | 82 | MSS | Sigmoid colon | II | FALSE |
| FAHNU-SC07 | FAHNU-SC | Male | 53 | MSS | Ascending colon | III | FALSE |
| FAHNU-SC08 | FAHNU-SC | Male | 70 | MSS | Rectum | III | FALSE |
| FAHNU-SC09 | FAHNU-SC | Female | 58 | MSS | Sigmoid colon | III | FALSE |
| FAHNU-SC10 | FAHNU-SC | Male | 76 | MSS | Rectum | II | FALSE |
| FAHNU-SC11 | FAHNU-SC | Male | 67 | MSS | Ascending colon | II | FALSE |
| FAHNU-SC12 | FAHNU-SC | Male | 59 | MSS | Ascending colon | III | FALSE |
| FAHNU-SC13 | FAHNU-SC | Male | 70 | MSS | Ascending colon | I | FALSE |
| FAHNU-SC14 | FAHNU-SC | Female | 82 | MSS | Ascending colon | III | FALSE |
| FAHNU-SC15 | FAHNU-SC | Male | 72 | MSS | Rectum | II | FALSE |
| FAHNU-SC16 | FAHNU-SC | Female | 44 | MSI | Rectum | III | FALSE |
| FAHNU-SC17 | FAHNU-SC | Male | 47 | MSS | Rectum | III | FALSE |
| FAHNU-SC18 | FAHNU-SC | Female | 78 | MSS | Sigmoid colon | III | FALSE |
| FAHNU-SC19 | FAHNU-SC | Male | 83 | MSS | Rectum | II | FALSE |
| FAHNU-SC20 | FAHNU-SC | Male | 58 | MSS | Sigmoid colon | III | FALSE |
| FAHNU-SC21 | FAHNU-SC | Male | 58 | MSS | Rectum | III | FALSE |
| FAHNU-SC22 | FAHNU-SC | Female | 48 | MSS | Rectum | I | FALSE |
| FAHNU-SC23 | FAHNU-SC | Male | 63 | MSS | Sigmoid colon | II | FALSE |
| FAHNU-SC24 | FAHNU-SC | Male | 63 | MSS | Ascending colon | II | FALSE |
| FAHNU-SC25 | FAHNU-SC | Female | 66 | MSI | Descending colon | III | FALSE |
| FAHNU-SC26 | FAHNU-SC | Female | 73 | MSS | Rectum | III | FALSE |
| FAHNU-SC27 | FAHNU-SC | Female | 66 | MSS | Caecum | III | FALSE |

NAT, Normal Adjacent Tissue.

**Table S2.** Stemness-related genes in colorectal cancer

| **Gene Symbol** | **PMID** |
| --- | --- |
| *ENPP3* | 26367378 |
| *MEX3A* | 35773527 |
| *LGR5* | 28358093 |
| *ASCL2* | 38050068/22637696/29425513 |
| *PTPRO* | 38050068 |
| *SOX4* | 33482915 |
| *SOX9* | 34571027 |
| *ZMYND8* | 33932349 |
| *CTNNB1* | 22637696 |
| *BMI1* | 34293363/22637696 |
| *ID1* | 22698403 |
| *ID3* | 22698403 |
| *PTCH1* | 29212028 |
| *USP22* | 34798260/30338942 |
| *ZRANB1* | 34798260 |
| *YAP1* | 26503053 |
| *SOX2* | 33953166 |
| *SEC62* | 33858476 |
| *OLFM4* | 19450592 |
| *BRG1* | 34415580 |
| *PROM1* | 34571027 |
| *CD44* | 18980968 |

**Table S3.** Signature gene sets of LGR5+ and LAPTM4B+ stem-like cells

| **LGR5^+^ Signature** | **LAPTM4B^+^ Signature** |
| --- | --- |
| *LGR5* | *LAPTM4B* |
| *OLFM4* | *TNNC2* |
| *TMEM19* | *EREG* |
| *TMEM238* | *C4orf48* |
| *CCL20* |  |
| *CES1* |  |
| *CPNE1* |  |
| *ARID3A* |  |
| *CCND2* |  |
| *ARPIN* |  |
| *CEL* |  |
| *CYFIP1* |  |
| *DEFB1* |  |
| *ENTPD5* |  |
| *FOXQ1* |  |
| *FZD5* |  |
| *GOLIM4* |  |
| *HMGN5* |  |
| *LONRF1* |  |
| *MAP3K20* |  |
| *MEP1A* |  |
| *NMT1* |  |
| *NR2F2* |  |
| *PDZD8* |  |
| *PRLR* |  |
| *RETNLB* |  |
| *RGMB* |  |
| *SERPINE2* |  |
| *SLC12A2* |  |
| *SLC9A3* |  |
| *TCEAL8* |  |
| *TNRC18* |  |
| *ZCCHC24* |  |
| *ZFC3H1* |  |

**Supplemental Methods**

**FAHNU-Bulk Cohort: DNA/RNA Extraction and Sequencing**

In this study, we conducted DNA and RNA sequencing on surgical resection samples from 148 patients with CRC recruited from Nanchang, China. The samples comprised both tumor tissues and peripheral blood mononuclear cells (PBMCs) from each patient. Total genomic DNA was extracted from the tumor tissues and PBMCs using the Genomic DNA Extraction Kit (Cat. No. QT-1001, IGENEBOOK). The extracted DNA was subsequently subjected to exome capture using the AIExome Enrichment Kit V3 (Cat. No. T600V1ST, iGeneTech), which targets exotic regions for sequencing. For RNA extraction, tumor tissues and adjacent normal tissues were processed using TRIzol reagent (Cat. No. RN0102, Aidlab). Immediately after RNA extraction, the samples were reverse-transcribed into complementary DNA (cDNA) to preserve RNA integrity and facilitate subsequent sequencing. All extracted DNA and synthesized cDNA samples underwent high-throughput sequencing on the MGISEQ-T7 platform (BGI, 150 PE).

**Spatial Transcriptome Spot Deconvolution**

Visium HD spatial transcriptomics data from 10x Genomics were obtained for three patients with CRC.^1^ Spot deconvolution was performed on the Visium HD data using default parameters of the RCTD (version 2.2.1), with reference to a previously constructed CRC single-cell atlas. The deconvolution results were integrated with the single-cell transcriptomic data using the Scanpy package, enabling comprehensive data visualization and analysis.

**Plasmid Construction and Lentivirus Production and Transduction**

In this study, molecular cloning techniques were utilized to insert shRNA sequences into a lentiviral vector. Specifically, the shRNA sequences targeting *LAPTM4B* were: shLAPTM4B-1:5’-gatcggatgatgtcatgtcagtgaatctcgagattcactgacatgacatcatctttttg-3’; shLAPTM4B-2:5’ gatcggatatgtgctatggctacttactcgagtaagtagccatagcacatatctttttg-3’. Single positive bacterial colonies were selected on LB agar plates containing ampicillin, and successful cloning confirmed by colony PCR and sequencing. HEK293T cells were cultured to package the lentivirus. The cell culture medium was harvested 48 hours post-transfection and filtered through a 0.45-micron filter. Transduction was carried out in SW480 and HT29 cell lines. Forty-eight hours post-transduction, the cells were subjected to puromycin selection to establish stably transduced cell populations. Quantitative reverse transcription PCR (qRT-PCR) and Western blot analysis were conducted to verify successful transduction. The primers used for qRT-PCR were: *LAPTM4B*-F: 5’-gctgtgtttggaactgctaccg-3’; *LAPTM4B*-R: 5’-gcagcaccattcacagtggcat-3’; *β-ACTIN*-F: 5’-cagggcgtgatggtgggcatg-3’; *β-ACTIN*-R: 5’-gtagaaggtgtggtgccagatt-3’. The antibodies used for Western blotting included LAPTM4B (Cat No. 18895-1-AP, Proteintech) and β-ACTIN (Cat No. EM21002, Huabio).

**Cell Culture**

Human CRC cell lines SW480 and HT29 were sourced from the Cell Bank of the Chinese Academy of Sciences (Shanghai, China). SW480 cells were cultured in Dulbecco’s Modified Eagle Medium (DMEM), while the HT29 cells were maintained in RPMI 1640 medium. Both cell lines were incubated at 37 °C in a humidified atmosphere with 5% CO₂. For tumor sphere culture, the cells were grown in ultra-low attachment culture dishes (Cat. No. 3471, Costar) using a CRC-specific conditioned medium, consisting of DMEM/F12 (Cat. No. 11320033, Gibco), 20 ng/ml EGF (Cat. No. 92701ES60, Yeasen), 20 ng/ml bFGF (Cat. No. 91330ES10, Yeasen), and B27 supplement (Cat. No. 60705ES10, Yeasen).

**Cell Proliferation Assay**

Tumor cells were cultured in 96-well plates to assess cell proliferation. The proliferation of SW480 and HT29 cells was measured using the Cell Counting Kit-8 (CCK-8, Cat. No. 96992, Sigma) and the 5-Ethynyl-2'-deoxyuridine (EdU) assay (Cat. No. C0075S, Beyotime), according to the manufacturers' instructions. The assays were performed strictly according to the provided protocols to ensure accurate measurements of tumor cell proliferation.

**Immunohistochemistry (IHC)**

IHC was conducted according to standard protocols. Tissue sections were incubated overnight at 4°C with an anti-LAPTM4B antibody (catalogue number 18895-1-AP, Proteintech). Subsequently, the sections were incubated at room temperature with a secondary antibody. Staining was visualized using a DAB (3,3'-diaminobenzidine) solution. A pathologist assessed the IHC score, considering both staining intensity and the proportion of positive cells. The IHC score was assessed by a pathologist, taking into account both the staining intensity and the proportion of positive cells.

**Fluorescence Multiplex Immunohistochemistry (mIHC)**

Fluorescence mIHC staining was conducted on FFPE samples using the Quadruple-Fluorescence immunohistochemical mouse/rabbit kit (Cat. No. RS0037, Immunoway). Briefly, FFPE sections were deparaffinised and underwent antigen retrieval in EDTA buffer (pH 9.0) under high-temperature conditions. The sections were then sequentially stained in four rounds with antibodies against EPCAM (Cat. No. EM1111, Huabio); LGR5 (Cat. No. TA502948S, Origene); LAPTM4B (Cat. No. 18895-1-AP, Proteintech); and Phospho-c-Myc (Cat. No. ET1609-64, Huabio). After staining, images of the specimens were captured using the TissueFAXS platform (TissueGnostics). Tumour tissue regions were identified and selected as regions of interest (ROIs) by a professional pathologist. These regions were subsequently subjected to quantitative analysis using the associated TissueQuest image analysis software.^2^

**Flow Cytometry**

SW480 and HT29 cell lines were cultured in a serum-free medium. After culture, cells were harvested and incubated with PE-conjugated anti-CD44 (Cat. No. 338807, Biolegend) and APC-conjugated anti-CD133 (Cat. No. 372805, Biolegend) antibodies for 30 minutes at room temperature. Following incubation, the cells were resuspended in PBS and analyzed using a flow cytometer (Beckman Coulter) to assess their staining profiles

**REFERENCES**

1. Oliveira MF, Romero JP, Chung M, et al. Characterization of immune cell populations in the tumor microenvironment of colorectal cancer using high definition spatial profiling. *BioRxiv*. 2024:2024.06. 04.597233.

2. Ecker RC, Steiner GE. Microscopy-based multicolor tissue cytometry at the single-cell level. *Cytometry A*. 2004;59(2):182-90.
